# Patterns of genetic variation and QTLs controlling grain traits in a collection of global wheat germplasm revealed by high-quality SNP markers

**DOI:** 10.1186/s12870-022-03844-x

**Published:** 2022-09-22

**Authors:** Chia-Hui Chou, Hsun-Shih Lin, Chen-Hsin Wen, Chih-Wei Tung

**Affiliations:** Department of Agronomy, National Taiwan University, No. 1, Sec. 4, Roosevelt Rd., Taipei, 10617 Taiwan

**Keywords:** Wheat, SNP array, Population structure, Linkage disequilibrium, Genome-wide association study (GWAS), Grain traits

## Abstract

**Background:**

Establish a molecular breeding program involved assembling a diverse germplasm collection and generating accurate genotypes to characterize their genetic potential and associate them with agronomic traits. In this study, we acquired over eight hundred wheat accessions from international gene banks and assessed their genetic relatedness using high-quality SNP genotypes. Understanding the scope of genomic variation in this collection allows the breeders to utilize the genetic resources efficiently while improving wheat yield and quality.

**Results:**

A wheat diversity panel comprising 39 durum wheat, 60 spelt wheat, and 765 bread wheat accessions was genotyped on iSelect 90 K wheat SNP arrays. A total of 57,398 SNP markers were mapped to IWGSC RefSeq v2.1 assembly, over 30,000 polymorphic SNPs in the A, B, D genomes were used to analyze population structure and diversity, the results revealed the separation of the three species and the differentiation of CIMMYT improved breeding lines and landraces or widely grown cultivars. In addition, several chromosomal regions under selection were detected. A subset of 280 bread wheat accessions was evaluated for grain traits, including grain length, width, surface area, and color. Genome-wide association studies (GWAS) revealed that several chromosomal regions were significantly linked to known quantitative trait loci (QTL) controlling grain-related traits. One of the SNP peaks at the end of chromosome 7A was in strong linkage disequilibrium (LD) with *WAPO-A1*, a gene that governs yield components.

**Conclusions:**

Here, the most updated and accurate physical positions of SNPs on 90 K genotyping array are provided for the first time. The diverse germplasm collection and associated genotypes are available for the wheat researchers to use in their molecular breeding program. We expect these resources to broaden the genetic basis of original breeding and pre-breeding materials and ultimately identify molecular markers associated with important agronomic traits which are evaluated in diverse environmental conditions.

**Supplementary Information:**

The online version contains supplementary material available at 10.1186/s12870-022-03844-x.

## Background

Wheat is the most widely grown cereal crop species in terms of cultivation area, ranking third in yield production and accounting for approximately 20% of the total daily calories and protein supply worldwide [1, 2]. Wheat provides important nutrients, including vitamins, dietary fiber, minerals, and phytochemicals, that are beneficial for human health [3]. The domestication of wheat occurred approximately 10,000 years ago. Specifically, two interspecific hybridization events were involved in the evolution of the modern hexaploid wheat *Triticum aestivum* (2n = 6x = 42, AABBDD). Cultivation and selection of wild emmer wheat led to the formation of the domesticated emmer wheat *T. turgidum* ssp. *dicoccum* (2n = 4x = 28, AABB), from which tetraploid durum wheat *T. turgidum ssp. durum* (2n = 4x = 28, AABB) evolved [4–6]. The origin of hexaploid spelt wheat (*T. aestivum Ssp. spelta*; 2n = 6x = 42, AABBDD) has been debated; it is not known whether spelt wheat is the ancestral form of hexaploid wheat or is derived from the hybridization of free-threshing hexaploid wheat and emmer wheat [7–9]. However, recent studies have suggested that spelt wheat emerged from the hybridization between hexaploid *T. aestivum* and emmer wheat [5, 6, 10].

The sequencing of the high-quality reference genome of the bread wheat cultivar Chinese Spring (International Wheat Genome Sequencing Consortium [IWGSC] RefSeq v1.0) [11] has enabled the genome-wide discovery of high-density markers, the study of the transcriptional landscape [12], the comparative analysis of structural variations [13], and the characterization of evolutionary history [6, 14, 15]. An improved genome sequence version, IWGSC RefSeq v2.1, was recently released. Sequencing gaps remaining in v1.0 were filled using PacBio long reads, and correction of scaffold orientation and ordering using a whole-genome optical map were achieved in this assembly [16]. With advancements in high-throughput genotyping technology, molecular marker discovery has accelerated. Detection of high-density single-nucleotide polymorphism (SNPs) via microarrays was applied to analyze the genome of tetraploid and hexaploid wheat species [17] and identify quantitative trait loci (QTLs) associated with agronomic traits [18–22], physiological traits [23], resistance to disease [24, 25], and resistance to preharvest sprouting [26].

Since the Green Revolution, significant breeding efforts have been made to increase wheat yields. Grain size and grain weight are components that determine yield potential, and several genes controlling grain-related traits have been identified and isolated via comparative genomics methods, including *TaGS5* [27], *TaGW7* [28], *TaGS3* [29], *TaCYP78A3* [30], and *TaWTG1* [31]. In recent years, genome-wide association studies (GWASs) have been applied to wheat to identify trait-controlling variants [22, 32–34] by exploiting the historic recombinant events that have accumulated over generations in wheat germplasms [35]. Several chromosomal regions associated with grain-related traits have been discovered in various wheat populations via association mapping approaches [22, 33, 36, 37]. Therefore, identifying QTL/genes or molecular markers associated with grain-related traits is a prerequisite when applying marker-assisted selection, especially for pyramiding beneficial alleles in elite cultivars, to improve wheat yield and quality.

In this study, a diversity panel consisting of 765 bread wheat, 60 spelt wheat, and 39 durum wheat accessions was genotyped using the Illumina iSelect wheat 90 K SNP array. The physical positions of array probe sequences were mapped to IWGSC RefSeq v2.1, which was released in early 2021. SNP quality, including call rate and call score, was evaluated by several criteria and compared with the SNP quality of the variants detected by next-generation sequencing (NGS). The population structure of this diverse wheat panel was assessed using SNPs with updated genomic positions, and a series of GWASs were then conducted to identify chromosomal regions associated with grain-related traits of bread wheat.

## Methods

### Plant material and SNP array genotyping

A wheat diversity panel comprising 39 durum wheat (*T. turgidum ssp. durum*), 60 spelt wheat (*T. aestivum ssp. spelta*), and 765 bread wheat (*T. aestivum ssp. aestivum*, spring wheat type) accessions were mainly obtained from the Wheat Germplasm Bank of the International Maize and Wheat Improvement Center (CIMMYT, Mexico) and the Germplasm Resources Information Network (GRIN) of the USDA-ARS (Supplementary Table S1). This collection included landraces (142), breeding materials (33), breeders lines (430), advanced/improved cultivars (20), cultivars (197), genetic material (2), wild material (1), and some with uncertain improvement status (40), the number of lines is indicated in parenthesis. Among four hundred and thirty breeders lines developed by CIMMYT’s wheat breeding program, three hundred and seventy lines have been evaluated in international and regional wheat trials such as Elite Selection Wheat Yield Trial (ESWYT, 24 lines), Semi-Arid Wheat Screening Nursery (SAWSN, 20 lines), High Rainfall Wheat Yield Trial (HRWYT, 12 lines), International Bread Wheat Screening Nursery (IBWSN, 44 lines), Fusarium Head Blight Screening Nursery (FHBSN, 98 lines), High Rainfall Wheat Screening Nursery (HRWSN, 126 lines), High Temperature Wheat Yield Trial (HTWYT, 46 lines).

The seeds used in this study were harvested from at least two-rounds of selfed propagation to ensure purity. The genomic DNA of each accession was extracted from fresh leaf tissue using a DNeasy 96 Plant Kit (Qiagen, Hilden, Germany) and checked for quality. The purified DNA was subsequently hybridized to an Illumina iSelect wheat 90 K SNP array, and array processing and fluorescent signal detection were performed according to the manufacturer’s protocol (Illumina, San Diego, USA).

### Assignment of the physical positions of iSelect 90 K SNP markers to the reference genome

The Illumina iSelect wheat 90 K SNP array involves 81,587 functional assays [17]. To obtain the physical positions of each SNP marker, the flanking sequences of the markers were obtained from this study [17] and searched against the content TRansposable Elements Platform (TREP) database (v2016) [38] with the following parameters: evalue 1e-10, best hit_score_edge 0.05, and best_hit_overhang 0.25. Markers highly similar to repetitive sequences were removed due to their difficult assignment to a specific chromosomal region.

The flanking sequences of the unique markers were mapped against the IWGSC RefSeq v1.0 [11] and IWGSC RefSeq v2.1 [16] assemblies of the bread wheat cultivar Chinese Spring via Basic Local Alignment Search Tool (BLAST). The parameters applied in the BLASTN algorithm were as follows: evalue 1e-10, best hit_score_edge 0.05, and best_hit_overhang 0.25. The chromosomal assignments of markers with multiple BLAST hits were determined based on the lowest E-value. The distribution of 90 K SNPs on the two assemblies was compared, the event involved in inconsistent SNPs orientation or order was defined by at least four adjacent markers, the interval sizes delimited by markers at distal ends were calculated in IWGSC RefSeq v2.1. Gene annotation of SNP was retrieved from IWGSC RefSeq Annotations (https://wheat-urgi.versailles.inra.fr/Seq-Repository/Annotations).

### Development of a SNP calling pipeline to genotype diverse wheat accessions

The image files of fluorescent signals, generated by the Illumina iSelect genotyping assays, were analyzed using the Polyploid Genotyping Module implemented in GenomeStudio v2.0.4 (Illumina, San Diego, USA). For each SNP marker, allele clustering was performed on the selected samples using the parameters included with the density-based spatial clustering of applications with noise (DBSCAN) clustering algorithm. The most important parameters were “cluster distance” and “minimum number of points in the cluster”. To determine the best combination of parameters, five cluster distances (0.02, 0.03, 0.05, 0.07, and 0.09) and three minimum numbers of points in clusters (5, 8, and 10) were tested and evaluated for their performance. Because the wheat accessions used in this study had undergone several generations of selfing, the “inbred population” option was selected because only two allelic clusters representing homozygous AA or BB groups were considered.

Determination of the best parameter combination was based on the sample call rate, sample p10 GC score (the 10th percentile of the distribution of GenCall scores for all SNPs), SNP call frequency, and SNP 10% GC score (the 10th percentile of the GenCall scores across all called genotypes). The genotype calls of Chinese Spring in our diversity panel were also used to evaluate SNP clustering performance. For markers with assigned physical positions, the corresponding genotypes at the same position in the Chinese Spring reference genome were extracted. The genotypes of Chinese Spring from our clustering results and the genotypes extracted from the reference genome sequence were then compared. Genotype calls (AA and BB) were converted to International Union of Pure and Applied Chemistry (IUPAC) nucleotide codes based on the information in Supplementary Table S5 in the study by [17]. Only SNPs assigned to unique physical positions were included in the final genotype table, which was subsequently converted to HapMap file format.

### Principal component analysis (PCA) and linkage disequilibrium (LD) analysis

SNP markers with a minor allele frequency (MAF) smaller than 0.01 and a missing percentage greater than 10% were removed. PCA was conducted in TASSEL v5.2.26 [39]. The software automatically converted the genotype data to numeric scores, and the missing data were imputed with the average score for each marker. Intrachromosomal LD was calculated using Plink v1.90 [40]. The window size for the calculation of LD was set to 25 Mb, and the LD between each pair of markers within the window was measured according to *r*^2^ values. To investigate LD decay in our diversity panel, the *r*^2^ values were plotted against the physical distance between each pair of markers, and a trend line was fitted using the Hill and Weir expectation of *r*^2^ [41], which was later modified by Remington et al. (2001) [42]. LD decay across the whole genome and within each subgenome was calculated for the three wheat species (durum wheat, spelt wheat, and bread wheat) in our dataset. The most commonly used threshold to declare no correlation between markers is an *r*^2^ of 0.1 or 0.2 [43]. For comparisons with the results of previous studies, values of LD decay using thresholds of *r*^2^ = 0.1 and 0.2 were reported.

### Model-based clustering analysis and genetic diversity index

The number of underlying subpopulations (K) in our diversity panel was determined using ADMIXTURE software [44]. The K value ranged from 1 to 12, and 10-fold cross-validation was performed. F_ST_ was calculated via GenoDive v3.04 to assess the genetic differentiation between subpopulations [45]. The mean pairwise difference (𝜋) for each subpopulation was calculated with TASSEL v5.2.56 [39]. We further detected genome-wide selection signals using BayeScan v2.1 [46] with the default parameter settings. A false discovery rate (FDR) < 0.05 was used as the threshold to identify significant SNPs.

### Genotyping-by-sequencing (GBS)

A subset consisting of 96 bread wheat lines from the diversity panel was subjected to GBS. The GBS library was prepared according to the protocol developed by Elshire et al. [47]. Briefly, genomic DNA was digested with ApeKI, followed by ligation of barcode sequences and common adapters. The barcoded samples were pooled and amplified by PCR, and the library was sequenced with a single-end length of 100 bp on an Illumina HiSeq 2500 platform. The quality of the sequence reads was assessed using FastQC v0.11.8 [48]. SNP calling was conducted via the TASSEL-GBS pipeline [49]. The reads were trimmed to 64 bp (not including the barcode) and subsequently mapped to the bread wheat reference genome IWGSC RefSeq v2.1 using Burrows-Wheeler Aligner (BWA) [50].

### Grain phenotyping and statistical analysis

The selected wheat accessions were grown over 2 years in the same experimental field. Within the same year, around 300 accessions were grown in the field with size around 0.1 ha, 18 individuals per accession were planted on one plot (plot size is 1 m × 1.2 m), two plots per accession were randomly arranged in the field. Mature grains of each accession were bulked, the dry and clean seeds (at least 100 grains and up to 600 grains per accession) harvested in two separate years (I and II) were scanned independently by an Epson Perfection V600 flatbed scanner at a 24-bit and 300 dpi resolution, a black cardboard box was used to cover the scanner to reduce internal reflection from the light emitted during scanning. The scanned image was saved as a JPG file for processing. Color calibration was performed by using the color Palette and histogram functions in Epson Perfection V600 Professional Mode. The grain size and color were measured from scanned images using GrainScan software developed by CSIRO [51]. The measurements included the area (mm^2^), perimeter (mm), grain length (mm), grain width (mm) and values representing three independent color channels. The value of each color channel was considered a proxy for the RGB color model. Pearson correlation coefficient was calculated between traits. Analysis of variance (ANOVA) for all the traits was performed using R. The broad-sense heritability was calculated as *H*^*2*^ = σ^2^_G_/(σ^2^_G_ + σ^2^_E_); where σ^2^_G_ was calculated as (MS_genotype −_ MS_residual_)/2 and σ^2^_E_ was MS_residual_.

### GWASs, local LD estimation and orthologous genes identification

Marker-trait associations were performed in TASSEL v5.2.56 [39]. The general linear model (GLM) estimates only SNP effects while controlling trait variation, and the mixed linear model (MLM) includes the centered identity-by-state (IBS) kinship matrix as a cofactor to reduce false-positive signals due to the relatedness among wheat accessions. Incorporating principal components (PCs) into the GLM and MLM could correct for the confounding effect caused by population structure. A total of four statistical models, the GLM, GLM + PC, MLM, and MLM + PC, were applied to estimate associations between markers and grain traits. The LD between peak SNP and neighboring SNPs was calculated and visualized using the R package “LDheatmap” [52].

The sequence of previously cloned rice genes controlling grain size traits (Supplementary Table S1 in [53]) was BLAST to search for wheat orthologs in LD block harboring peak SNP. For wheat orthologs identification, the coding sequence (CDS) of the cloned rice genes annotated to grain morphological traits in the Q-TARO database [54] were retrieved from the RAP-DB database [55] and BLAST against the wheat genome assembly IWGSC RefSeq v2.1 using the parameters: evalue 1e-10, best hit_score_edge 0.05, and best_hit_overhang 0.25.

## Results

### Anchoring the Illumina iSelect wheat 90 K markers to IWGSC RefSeq v1.0 and v2.1

The Illumina iSelect wheat 90 K array includes 81,587 SNP detection assays corresponding to 517,587 hybridization sites in the wheat genome. The flanking sequences of each assay were obtained from a previous study [17], and 277 assays showing high similarity to repetitive sequences in the TREP database were removed. The remaining ~ 81,000 assay sequences were searched against the IWGSC RefSeq v1.0 and v2.1 assemblies using the BLAST, which yielded 242,632 and 380,430 hits, respectively. Excluding the assays without any hits, assays with a single hit or with hits with the lowest E-value were selected, which resulted in 57,851 markers (together named as “90K_Refv1”), and 57,398 markers (together named as “90K_Refv2”) (Table 1). A similar marker distribution was observed in the two datasets, and a reduced number of total markers and markers assigned as “unknown” in IWGSC RefSeq v2.1 that the genotype call accuracy had improved. Comparing the two datasets (90K_Refv1 and 90K_Refv2), we found that 56,597 markers (98.98%) were commonly assigned to the same chromosomes, while 583 markers (1.02%) were mapped to different chromosomes in the two genome assemblies. Gene annotation of 56,597 SNP markers was retrieved from IWGSC RefSeq v2.1, 50,006 markers were located in the genic region, 901 and 1666 markers were within 2 kb upstream and downstream of annotated gene, respectively (Supplementary Table S2). Most of the inconsistent chromosomal assignments involved markers that were assigned to the unknown chromosome in IWGSC RefSeq v1.0 but assigned to real chromosomes in IWGSC RefSeq v2.1 (417, 71.53%), and the second most occurred within homoeologous groups (104, 17.84%) (Supplementary Fig. S1). Only 22 markers (3.77%) were mapped to the real chromosomes in IWGSC RefSeq v1.0 but were mapped to the unknown chromosome in IWGSC RefSeq v2.1. When analyzing the order and orientation of markers on the same chromosome between IWGSC RefSeq v1.0 and v2.1, we detected 63 events that ranged from 1.03 kb to 2.46 Mb in size, which could be the result of contig orientation errors (Supplementary Fig. S2). Eleven events ranging from 1.20 to 329.76 Mb in size were related to a contig ordering problem. Eighteen events ranging from 652.18 kb to 550.38 Mb were due to problematic orientation and ordering of contigs. The physical positions of array markers in IWGSC RefSeq v1.0 and v2.1 are provided in Supplementary Table S2. A total of 44,803 markers anchored to IWGSC RefSeq v1.0 were also reported in a previous study [56], and we found that the genomic positions of 43,642 markers (97.41%) were in agreement (Supplementary Table S2).

**Table 1:** Number of Illumina iSelect 90 K SNP markers assigned to wheat chromosomes according to two IWGSC RefSeq genome assemblies

Homoeologous group	90K_Refv1				90K_Refv2			
	Subgenome			Total	Subgenome			Total
	A	B	D		A	B	D	
1	2802	3340	2373	8515	2804	3319	2361	8484
2	3299	4718	2834	10,851	3323	4682	2827	10,832
3	2696	3060	2007	7763	2686	3078	2015	7779
4	2421	2172	1427	6020	2424	2176	1473	6073
5	2849	3546	2307	8702	2837	3515	2308	8660
6	2614	2796	1810	7220	2616	2786	1864	7266
7	3099	2665	2261	8025	3096	2676	2267	8039
Total	19,780	22,297	15,019	57,096	19,786	22,232	15,115	57,133
Unknown	755			57,851	265			57,398

### Determine SNP genotypes for 864 wheat accessions

**Fig. 1:**
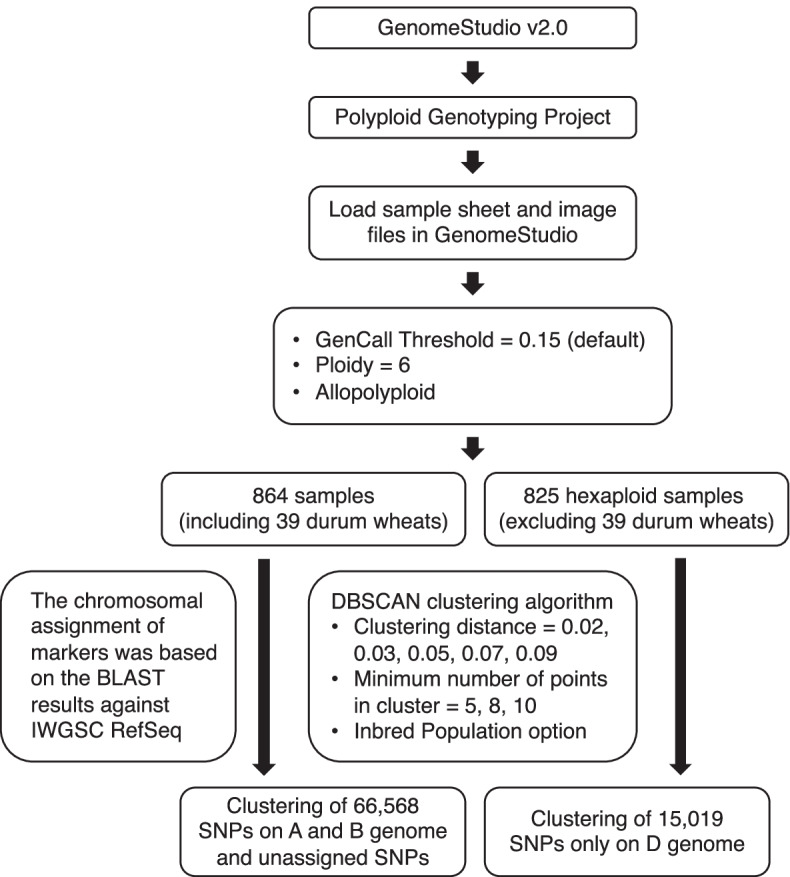
In-house pipeline developed for clustering SNPs in wheat subgenomes. Each box contains information describing the functionality, parameters, input samples and output results

To determine accurate SNP genotypes for each wheat accession, we developed a customized SNP clustering pipeline based on Illumina GenomeStudio Software v2.0.4 (Fig. 1). Within the GenomeStudio polyploid genotyping module, we applied the DBSCAN algorithm to cluster the samples. By comparing the number of samples or the number of markers meeting the specific threshold of the call rate (0.9) and call score (0.4) (the result of each threshold was presented in separate sheet in Supplementary Tables S3), we determined that the best setting to generate high-quality genotypic information was a cluster distance of 0.05 and minimum number of points in a cluster of 5 for markers in the A and B genomes as well as a cluster distance of 0.07 and minimum number of points in a cluster of 5 for SNPs in the D genome. To evaluate the accuracy of SNPs detected by the 90 K array, we sequenced, via genotyping-by-sequencing (GBS), the genome of 96 bread wheat accessions, including that of the Chinese Spring variety. After filtering the low-coverage reads, 12,763,672 unique sequences were aligned to IWGSC RefSeq v2.1, and variants were identified using the TASSEL-GBS pipeline [49]. Comparing the genotype calls of the 96 wheat accessions obtained from GBS and 90 K SNP array detection, at shared loci without missing values, we found that 97.98% was in agreement (Table 2 and Supplementary Table S4).

**Table 2:** Consistency of genotype calls between the GBS and 90 K arrays of 96 wheat accessions. The SNPs with MAF > 0.01 and missing rate < 20% were used for comparison

Chromosome	Number and missing rate of 90K_Refv2 SNPs	Number and missing rate of GBS SNPs	Number and agreement percentage of shared markers
1A	1510 (0.34%)	473 (11.20%)	0
1B	1883 (0.27%)	548 (11.45%)	2 (100%)
1D	847 (0.26%)	271 (10.56%)	0
2A	1585 (0.16%)	662 (11.33%)	0
2B	2269 (0.24%)	1023 (11.10%)	6 (98.65%)
2D	882 (0.15%)	287 (11.13%)	1 (100%)
3A	1302 (0.13%)	555 (10.95%)	4 (92.39%)
3B	1659 (0.18%)	893 (11.03%)	5 (100%)
3D	504 (0.17%)	382 (11.33%)	0
4A	1138 (0.21%)	448 (11.50%)	0
4B	917 (0.10%)	318 (11.52%)	3 (100%)
4D	347 (0.12%)	99 (12.14%)	0
5A	1430 (0.24%)	572 (11.00%)	8 (99.58%)
5B	1823 (0.12%)	784 (10.89%)	6 (99.81%)
5D	455 (0.07%)	131 (11.07%)	0
6A	1461 (0.18%)	504 (11.08%)	5 (92.06%)
6B	1542 (0.38%)	870 (11.43%)	2 (100%)
6D	547 (0.28%)	227 (11.35%)	2 (97.59%)
7A	1733 (0.29%)	873 (11.02%)	1 (100%)
7B	1494 (0.24%)	935 (11.28%)	1 (98.81%)
7D	522 (0.23%)	257 (11.24%)	0
Unknown	163 (0.51%)	102 (10.61%)	0
Overall	26,013 (0.22%)	11,214 (11.17%)	46 (97.98%)

### Population structure, selection signal and LD in wheat species and improved breeding lines

**Fig. 2:**
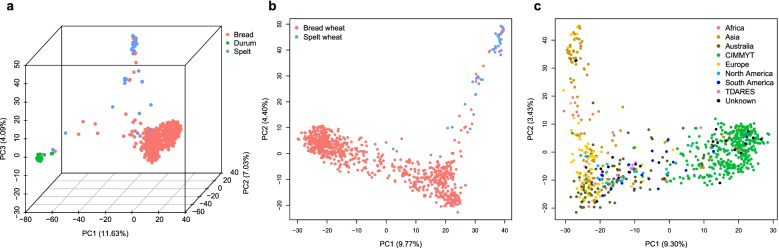
PCA of the diversity panel. **a** 3D scatter plot of the first three PCs representing 864 wheat accessions based on 28,836 SNPs in the A and B genomes. **b** Distribution of 825 hexaploid wheat accessions on the basis of 31,854 SNPs in the A, B, and D genomes. **c** Distribution of the 765 bread wheat accessions on the basis of 29,803 SNPs in the A, B, and D genomes. The proportion of the total variance explained by each PC is shown on the axis label. The color of each point represents the species or origin of accession

After removing SNPs with MAF < 0.01 and missing rate > 10%, a total of 28,836 polymorphic SNPs in the A and B genomes were used to analyze the population structure of 864 tetraploid and hexaploid wheat germplasms. The first PC separated tetraploid durum wheat from hexaploid wheat; bread wheat and spelt wheat were further differentiated by other PCs (Fig. 2a).

When the polymorphic markers in the D genome were included, which resulted in total 31,854 polymorphic SNPs in the A, B and D genomes, to analyze the population of 825 hexaploid wheat accessions, the results showed that the first two PCs could differentiate spelt wheat from bread wheat and could differentiate bread wheat accessions (Fig. 2b). To better characterize the 765 bread wheat accessions, the origin of genotypes was retrieved from the GRIN database (https://npgsweb.ars-grin.gov/gringlobal/search). Interestingly, the accessions developed at CIMMYT were grouped together and separated from others by PC1 based on 29,803 polymorphic SNPs (Fig. 2c). The accessions separated by PC2 were somewhat correlated with geographical origin, and one group of accessions collected in Asia was genetically differentiated from the accessions collected in Europe, Australia, and America.

**Fig. 3:**
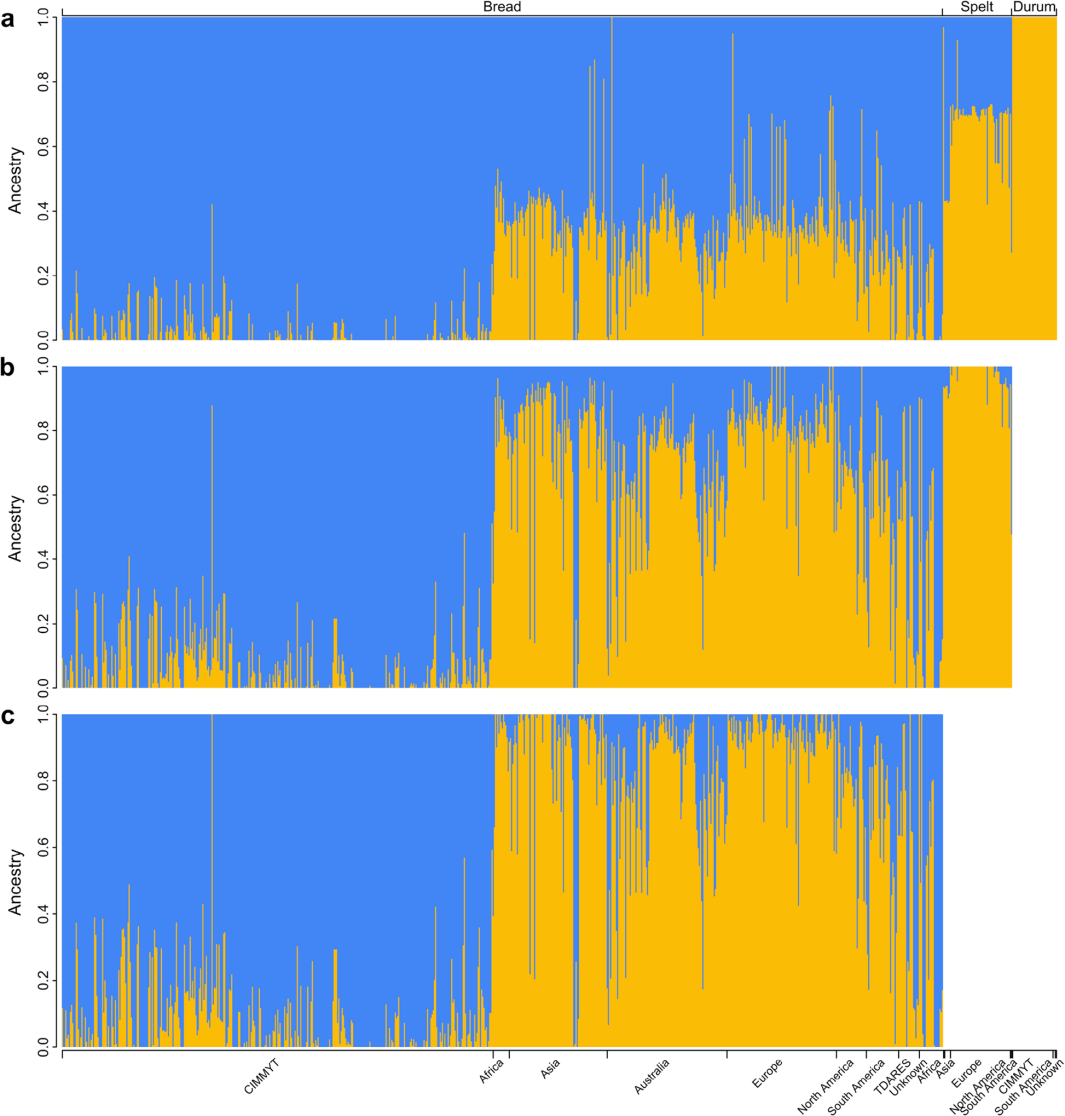
ADMIXTURE ancestry coefficient (K = 2) for diverse wheat accessions. **a** Eight hundred sixty-four durum wheat, spelt wheat and bread wheat samples analyzed by SNP markers on the A and B genomes. **b** Eight hundred twenty-five hexaploid wheat samples analyzed by markers on the A, B, and D genomes. **c** Seven hundred sixty-five bread wheat samples analyzed by markers on the A, B, and D genomes. Each stacked bar represents one genotype, and the color and length are proportional to the ancestral components. The origin of the accessions is denoted below each stacked bar

To characterize the genetic ancestry of various wheat accessions, the model-based clustering software ADMIXTURE was applied to calculate an individual accessions’ ancestry coefficient. By the use of SNPs in the A and B genomes, the majority of durum wheat, spelt wheat, and bread wheat accessions could be clustered into species-specific groups (Fig. 3a). Some samples had mixed ancestral compositions, suggesting that gene flow or introgression events had occurred. Ancestry analysis of 825 hexaploid wheat and 765 bread wheat accessions using markers on A, B, and D genome separated cultivars developed at CIMMYT from the rest of the accessions at K = 2 (Fig. 3b and c). The CIMMYT germplasms shared a high proportion of ancestral components, which was in agreement with the PCA results (Fig. 3c). To assess the level of subpopulation differentiation (via a fixation index [F_ST_]) within the bread wheat group, individual accession was assigned to a specific subpopulation when the proportion of a single ancestry was larger than 0.8, a total of 259 individual accessions were assigned to subpopulation Pop1 (red dots), 354 genotypes belonged to subpopulation Pop2 (blue dots), and 152 accessions were considered admixtures (Supplementary Fig. S3). Pop1 mainly consisted landraces (119 accessions) and cultivars (110 accessions) from worldwide countries, Pop2 is dominated by 339 breeders lines. The pairwise F_ST_ between Pop1 and Pop2 was 0.201, suggesting moderate differentiation (Table 3). The mean pairwise difference (𝜋) of Pop1 was 0.30, while that of Pop2 was only 0.21. Although Pop2 comprised more accessions than Pop1 did, the mean pairwise difference in Pop2 was lower than that in Pop1, which could result from 92.09% of the accessions in Pop2 being improved lines developed by CIMMYT, and many of them shared the same parents or have similar pedigree history (Supplementary Table S1).

**Table 3:** F_ST_ value between subpopulations within the bread wheat group

Population	Pop1	Pop2	Admixed
**Pop1**	0.000	0.201	0.077
**Pop2**	0.201	0.000	0.099
**Admixed**	0.077	0.099	0.000

We applied BayeScan v2.1 [46] to detect genome-wide selection signals. Our results showed that 88 SNPs with an FDR < 0.05 significantly differentiated between Pop1 (cultivars or landraces) and Pop2 (CIMMYT improved germplasms). The F_ST_ values of these significant signals ranged from 0.32870 to 0.42347 (Table 4 and Supplementary Table S5), and the SNPs were located on chromosomes 1A, 1B, 1D, 3A, 3B, 4A, 4B, 4D, 5D, 6A, and 6D (Supplementary Fig. S4). A cluster of significant SNPs was identified on chromosome 1A in the region between 345 Mb and 377.5 Mb, suggesting that this region might have been subject to strong selection. All the significant loci had a positive alpha value, suggesting that these loci were under diversifying selection.

**Table 4:** Results of Bayescan showing putative genomic regions differentiated between Pop1 and Pop2

Marker Name	Chr	RefSeqv2.1 (bp)	Fst	qval	Gene annotation
BS00086680_51	1A	283,434,503	0.42347	0.0027169	TraesCS1A03G0427800
wsnp_Ex_c14866_22995097	1A	344,966,381	0.40759	0.0025669	TraesCS1A03G0509200LC
wsnp_Ex_c1374_2630830	1A	353,665,720	0.38302	0.0058256	TraesCS1A03G0524200
wsnp_Ex_c8885_14842394	1A	353,701,188	0.38325	0.0073579	TraesCS1A03G0524400
wsnp_Ra_rep_c74936_72685894	1A	354,778,944	0.38203	0.0070857	TraesCS1A03G0525100
IACX2325	1A	357,984,644	0.38942	0.0031781	TraesCS1A03G0531100
wsnp_Ex_c3906_7086162	1A	370,238,544	0.37882	0.0082217	TraesCS1A03G0547700
BS00081455_51	1A	371,510,813	0.40244	0.0027169	TraesCS1A03G0551300
wsnp_Ex_c4605_8241260	1A	371,519,074	0.40171	0.0026253	TraesCS1A03G0551400
Kukri_c54467_100	1A	377,521,899	0.37488	0.009663	TraesCS1A03G0560100
Ra_c11488_297	1B	58,403,113	0.36423	0.020924	TraesCS1B03G0172700
wsnp_Ex_c14832_22953906	1B	58,553,627	0.36382	0.019809	TraesCS1B03G0173000
wsnp_Ex_c33654_42106735	1B	60,378,596	0.35842	0.022868	TraesCS1B03G0176700
wsnp_Ex_c41969_48673442	1B	403,457,802	0.3782	0.0085049	TraesCS1B03G0637300
D_contig04348_649	1D	296,218,439	0.37476	0.011175	TraesCS1D03G0523400
BS00021878_51	1D	300,171,366	0.37815	0.0076462	TraesCS1D03G0531700
Excalibur_c27055_1326	3A	486,468,087	0.33126	0.048531	TraesCS3A03G0650400
Tdurum_contig31586_197	3A	512,355,527	0.36841	0.012207	TraesCS3A03G0689300
wsnp_Ex_c2580_4800249	3B	470,056,794	0.33203	0.045641	TraesCS3B03G0734200
wsnp_Ex_c34975_43204180	3B	471,012,861	0.3287	0.049573	TraesCS3B03G0735500
Kukri_c18009_398	3B	475,225,424	0.33396	0.041674	TraesCS3B03G0741000
TA002241–1114	3B	501,573,733	0.35778	0.017918	TraesCS3B03G0781500
wsnp_Ex_c5378_9505533	3B	501,573,733	0.35871	0.019232	TraesCS3B03G0781500
BS00037898_51	3B	501,926,217	0.36162	0.016595	TraesCS3B03G0782700
Tdurum_contig75784_771	3B	502,260,476	0.36358	0.015299	TraesCS3B03G0783000
BS00047274_51	3B	642,665,525	0.34594	0.025818	TraesCS3B03G0992500
Ra_c106076_67	3B	644,913,215	0.34285	0.028626	TraesCS3B03G0995200
wsnp_Ex_c8825_14757625	3B	645,214,868	0.33913	0.03614	TraesCS3B03G0995600
BS00076457_51	3B	820,219,334	0.34512	0.02964	TraesCS3B03G1427000LC
Tdurum_contig19376_810	4A	28,573,747	0.37333	0.011871	TraesCS4A03G0067900
Ex_c40210_281	4A	56,468,205	0.36411	0.015733	TraesCS4A03G0119700
wsnp_BE591195A_Ta_1_1	4A	71,226,338	0.40432	0.0025003	TraesCS4A03G0146700
wsnp_Ex_c7011_12080274	4A	146,300,496	0.39682	0.0034903	TraesCS4A03G0243200
wsnp_Ra_rep_c107017_90667618	4A	165,449,299	0.38209	0.0079182	TraesCS4A03G0272200
RAC875_c110384_153	4A	212,332,496	0.3819	0.0051577	TraesCS4A03G0309400
wsnp_Ex_c10186_16720660	4A	233,713,164	0.38307	0.0064729	TraesCS4A03G0324600
wsnp_CAP7_c2931_1395666	4A	464,533,298	0.38406	0.0055006	TraesCS4A03G0503100
wsnp_Ex_c5979_10480527	4A	466,419,006	0.41374	0.0024002	TraesCS4A03G0507300LC
wsnp_Ex_rep_c70327_69270561	4A	488,165,445	0.35563	0.023532	TraesCS4A03G0536300
wsnp_Ku_c5979_10559245	4A	521,249,651	0.35917	0.020375	TraesCS4A03G0585900
Kukri_c74651_223	4A	533,271,898	0.34889	0.026759	TraesCS4A03G0601800
wsnp_Ex_rep_c67779_66463916	4A	533,582,113	0.3376	0.040703	TraesCS4A03G0602200
Excalibur_c31814_298	4A	533,820,364	0.35778	0.0222	TraesCS4A03G0602300
IAAV7636	4A	534,460,817	0.34136	0.037012	TraesCS4A03G0603500
IAAV971	4B	43,533,249	0.38945	0.010837	TraesCS4B03G0112000
Excalibur_c56787_95	4B	62,313,549	0.39457	0.0088047	TraesCS4B03G0145400
Excalibur_c17607_542	4B	81,062,793	0.38883	0.011518	TraesCS4B03G0183100
wsnp_RFL_Contig4151_4728831	4B	183,053,308	0.38448	0.01486	TraesCS4B03G0318300
RAC875_c101563_102	4B	212,025,810	0.38659	0.013955	N/A
Excalibur_c55414_216	4B	242,295,896	0.34019	0.037864	TraesCS4B03G0376700LC
RAC875_c46966_193	4B	242,296,560	0.34716	0.025007	N/A
RAC875_c75075_313	4B	310,932,752	0.35545	0.031596	TraesCS4B03G0428900
RAC875_c12495_1391	4B	362,697,044	0.38983	0.013499	TraesCS4B03G0469400
BobWhite_c9876_331	4B	375,327,326	0.34685	0.039731	N/A
wsnp_JD_c1549_2185341	4B	389,540,610	0.34203	0.047573	TraesCS4B03G0502100
wsnp_Ex_c25373_34639805	4B	481,850,675	0.3663	0.024242	TraesCS4B03G0630800LC
RAC875_c107130_384	4B	648,735,419	0.36646	0.012654	TraesCS4B03G0930800
Kukri_c7791_99	4D	4,232,342	0.38036	0.018765	TraesCS4D03G0014600
Kukri_c35140_75	4D	208,791,961	0.38259	0.017043	TraesCS4D03G0351700
wsnp_Ra_c9233_15459255	5D	129,165,508	0.3649	0.035251	TraesCS5D03G0264200
RAC875_rep_c70595_321	5D	155,195,977	0.36619	0.032533	TraesCS5D03G0282500
Excalibur_c15835_86	5D	393,060,408	0.3838	0.0043588	TraesCS5D03G0666300
IAAV6265	5D	400,879,728	0.41294	0.0039277	TraesCS5D03G0684600
BobWhite_c27364_296	6A	616,983,067	0.34329	0.0277	TraesCS6A03G1028700
IAAV8527	6D	410,996,114	0.36902	0.018337	TraesCS6D03G0670800
Kukri_c31995_1948	6D	423,456,552	0.37339	0.014411	TraesCS6D03G0689000
wsnp_Ex_c1690_3206784	6D	427,650,981	0.37669	0.013081	TraesCS6D03G0696600
wsnp_Ra_c13881_21836489	6D	430,642,760	0.35451	0.021543	TraesCS6D03G0701400

**Fig. 4:**
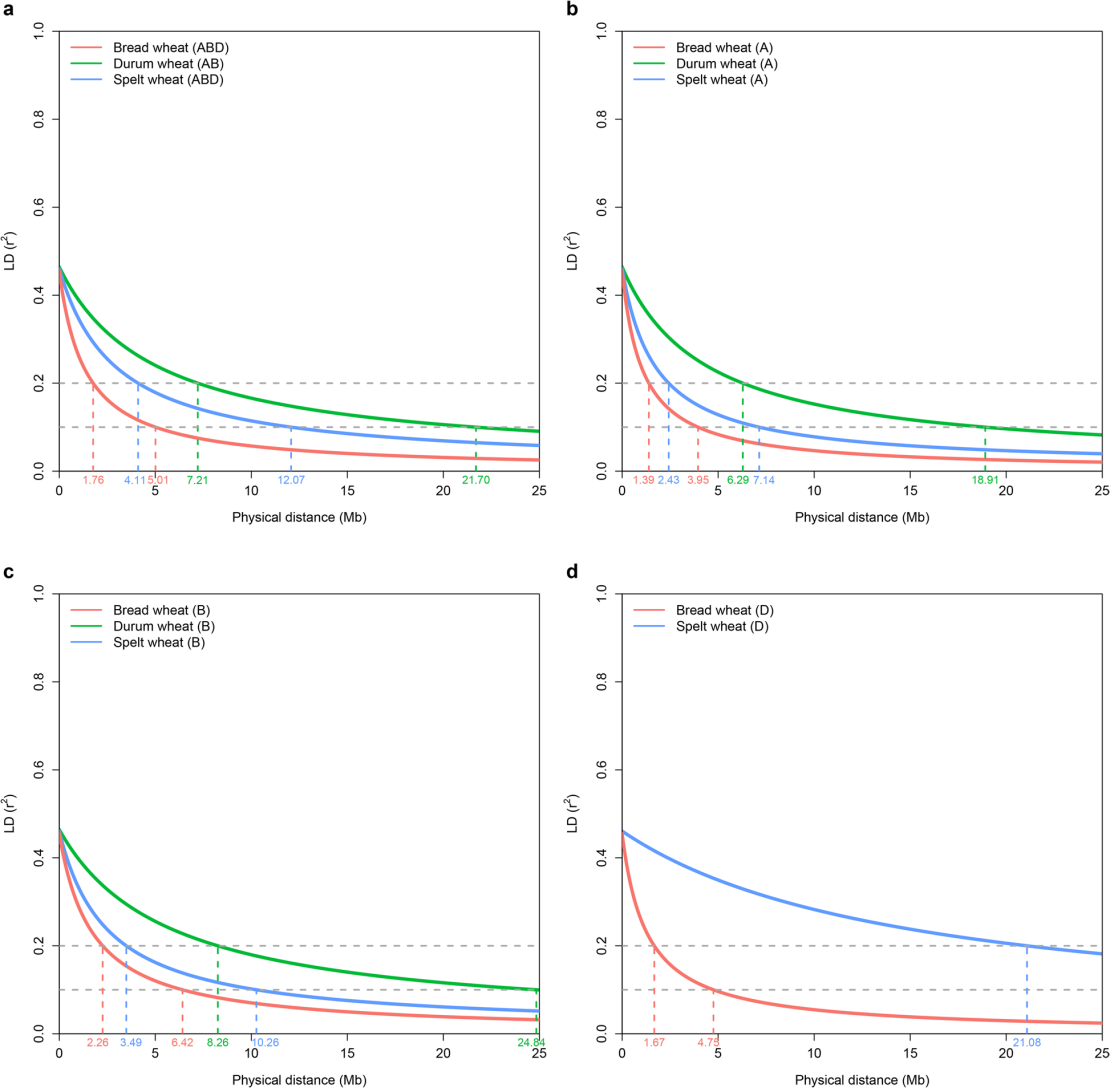
LD of bread wheat, spelt wheat, and durum wheat. **a** Genome-wide LD decay of three wheat species. **b** LD decay of subgenome A of the three species. **c** LD decay of subgenome B of the three species. **d** LD decay of subgenome D of spelt wheat and bread wheat. The physical distance (in megabases) is plotted against the LD estimate (*r*^2^) for pairs of markers

LD was analyzed in durum wheat, spelt wheat, and bread wheat separately. Among the three species, bread wheat exhibited the fastest genome-wide LD decay, and the physical distances at which genome-wide LD (*r*^2^) decayed from the initial value of ~ 0.45 to 0.2 were 1.76 Mb in bread wheat, 4.11 Mb in spelt wheat, 7.21 Mb in durum wheat (Fig. 4a). When comparing the decay of subgenome LD among the three species, we found that the LD of subgenomes A and B was greatest in durum wheat, and the highest extent of LD was detected in the D genome (*r*^2^ = 0.2 at 21.08 Mb) in spelt wheat (Fig. 4b-d). LD in Pop 1 (cultivars or landraces) decayed faster than that in the Pop 2 and admixture groups when the SNPs in the A or B genome or three genomes together were analyzed (Supplementary Fig. S5); however, a long range of LD was observed in Pop 1 when the D genome alone was analyzed, followed by the Pop 2 and admixture populations.

### GWASs revealed chromosomal regions controlling grain-related traits

**Fig. 5:**
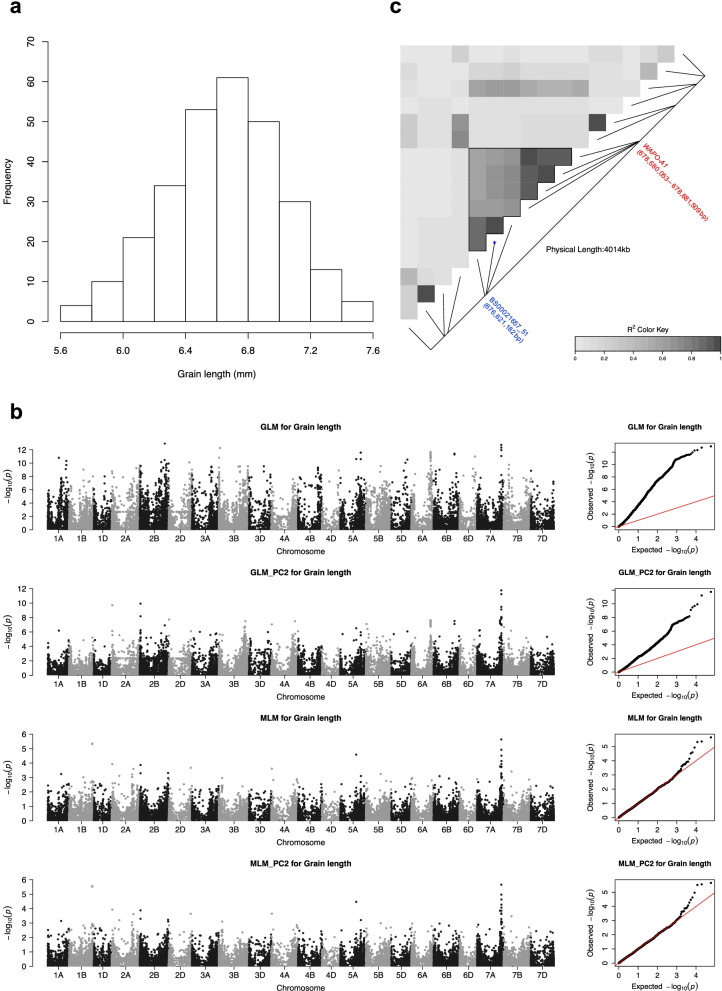
Genome-wide association mapping for grain length. **a** Distribution of the grain length of 280 bread wheat accessions. **b** Manhattan plot of the four models (GLM, GLM_PC, MLM, MLM_PC) and associated quantile-quantile (Q-Q) plot representing the statistical association between each SNP and grain length. **c** The pairwise LD of SNPs surrounding the peak marker on chromosome 7A was calculated. The position of peak marker BS00021657_51 and WAPO-A1 is labeled

To validate the efficacy of high-quality SNPs genotyped in diverse accessions, we performed genome-wide association study using grain-related traits known for their high heritability characteristics. Analyzing 2 years of grain phenotypic data in 280 wheat accessions showed grain trait across years (I and II) was highly correlated (i.e., grain length is 0.83, grain color is 0.86), the broad-sense heritability of each trait was then calculated, which ranged from 0.62 to 0.86 (Supplementary Table S6). A total of 29,467 polymorphic SNPs with MAF greater than 0.01 and a missing percentage of less than 10% were used to identify genotypic associations with grain-related traits in 280 bread wheat accessions. A Manhattan plot and Q-Q plot generated from the statistical models for each grain trait are shown in Fig. 5b and Supplementary Figs. S6, S7, S8, S9, S10 and S11, the *p-value* of significant SNPs smaller than 10^− 6^ or 10^− 4^ detected in GLM or MLM model and their associated R^2^ for each grain trait was presented in Supplementary Table S7. For the normally distributed grain length trait, a major SNP peak was identified at the end of chromosome 7A across all analytical models (Fig. 5a and b), suggesting that this SNP peak is adjacent to a QTL for grain length. Interestingly, this region was also significantly associated with grain surface area and grain perimeter (Supplementary Figs. S7 and S8). This candidate region was further refined by analyzing the local LD between the peak marker (BS00021657_51) and neighboring markers spanning a 10 Mb region, and the results suggested that the interval was located between 676,603,251 and 678,873,631 bp on chromosome 7A (Fig. 5c). Wheat chromosome 7A is known to harbor several genes related to grain traits, including *TaWTG1* [31], *TaGASR7-A1* [57], *TaTGW-7A* [58], *TaTEF-7A* [59], and *WAPO-A1* [60]. Among these genes, *WAPO-A1* was located within the region identified in this study. *WAPO-A1* was previously identified as a candidate gene involved in spikelet number per spike, but additional experiments are needed to determine whether *WAPO-A1* has a pleotropic effect or whether there is a novel gene that regulates grain length. For grain color, one significant SNP cluster located at the end of long arm of chromosome 3B was commonly detected in three color channels across all models examined (Supplementary Figs. S9, 10 and S11), the significant SNP (Excalibur_rep_c97324_623) at 771,937,474 bp was previously reported to associate with grain color in the U.S winter wheat [61].

Grain size-related traits have been investigated extensively in rice. Therefore, we extracted the sequences from functionally characterized rice genes known to control seed morphological traits from Q-TARO database [54] and a review paper [53] to search for the wheat orthologs, the identified orthologs (total 191) were then mapped to our GWAS detected regions. The results showed that TraesCS3A03G0430300 on chromosome 3A, the ortholog of rice grain-size associated gene *Rdd1* [62] was near a significant SNP wsnp_Ex_c1538_2937905 controlling grain length (Supplementary Table S8). The orthologs of rice grain length controlling genes *GL3* and *OsPPKL3* [63] were located on wheat chromosome 5A and 5D. Three significant SNPs, Kukri_c28080_887 and RAC875_rep_c112205_166 associated with grain perimeter and BS00073670_51 for grain length, were located near TraesCS5A03G0897900 on chromosome 5A. SNP IAAV9053 was overlapped with TraesCS5D03G0859400 on chromosome 5D. Other significant SNPs such as wsnp_Ex_c1538_2937905 on chromosome 3A, BS00022800_51 on chromosome 3B, and Tdurum_contig11827_678 on chromosome 7B, were colocalized with wheat orthologs of rice genes controlling grain morphological traits (Supplementary Table S8).

## Discussion

### Identification of the physical positions of 90 K SNP markers on the IWGSC RefSeq v2.1 assembly

Seven years since the release of the Illumina iSelect wheat 90 K SNP array to the public [17], more than one thousand articles have cited the original publication, which suggested that a substantial number of research experiments have been conducted via this genotyping array. With the recent release of the IWGSC RefSeq v2.1 assembly [16], it is necessary to update the physical position of molecular markers to the current version. In this study, the flanking sequences of 90 K SNP markers were mapped to the IWGSC RefSeq v1.0 and v2.1 independently, and most mapped markers were located on the B genome, followed by the A genome and D genome, which is consistent with previous findings [17, 64]. Thousands of SNPs located in problematic contigs were corrected, and their genomic positions are now accurate. Moreover, 279 previously unplaced scaffolds (74.96 Mb) were anchored onto pseudomolecules, which reduced the number of array markers assigned to the unknown chromosome. These refinements contributed to the improved sequence and assembly quality of IWGSC RefSeq v2.1 [16].

### Controlling the accuracy of array-anchored genotypes

Fluorescence intensity generated by hybridization between genomic DNA and array probes is the source of allele clustering, which often requires manual curation for error-prone SNPs in polyploid species [17, 65]. It is extremely time consuming and challenging to manually inspect SNP clusters without predefined cluster files, especially for diverse wheat accessions. In this study, we developed a customized allele clustering pipeline to avoid manual curation, and several indices were used to evaluate the clustering performance. In general, a larger cluster distance and a smaller minimum number of points in the cluster lead to a higher number of samples and SNPs with call rates > 0.9. Compared to cluster distance, the minimum number of points showed variable effects on genotype quality. Therefore, we selected an optimal cluster distance to obtain superior clustering performance, resulting in a relatively high call rate and call score.

Array-based genotyping has been compared to GBS in various contexts [56, 66, 67]. In this study, the genotypes detected at shared loci from two platforms were highly concordant, which supported the ability of our in-house SNP calling pipeline to generate accurate genotype sequences. Array-based systems provide a unique advantage for easy integration of genotypes from samples processed in different laboratories, and the fixed genomic positions of assayed markers could help streamline downstream applications, such as comparing detected QTLs for the same traits and developing trait-associated markers for selection. It is also important to consider the tradeoff between the cost of genotyping and bioinformatic infrastructure establishment when deciding which genotyping platform to use.

### Population structure and differentiation of diverse wheat accessions

When SNP genotypes of the A and B genomes were used, PCA could easily separate our diversity panel into three main clusters corresponding to bread wheat, spelt wheat, and durum wheat, which was in agreement with the results from phylogenetic and ADMXITURE analyses. In the analysis, some accessions were placed between defined clusters, and the admixed accessions might be the result of gene flow between wheat species. Complex historical events of hybridization have led to frequent gene flow or introgression between wheat species and their wild relatives [6, 14]. Using SNPs in the A, B, and D genomes to characterize the population structure within 765 bread wheat lines, we found that the improved wheat lines developed at CIMMYT were separated from accessions collected in other countries. A recent study analyzed the diversity of 56,342 domesticated hexaploids, including landraces, widely grown cultivars, elite breeding lines and nursery germplasm, from CIMMYT [68]. The authors found that a large group of elite materials clearly differentiated from landraces and genetic stocks, those elite lines had the varieties “Kauz”, “Pastor” and “Veery” in their pedigree history, apparently, these three varieties were also found in 31% of CIMMYT breeders lines of our collection, Kauz and Pastor were appeared in 97 and 68 records respectively (Supplementary Table S1). Improved varieties developed by CIMMYT wheat breeding program could have undergone different levels of artificial selection, and the shared parentages could cause the allele frequency of these accessions to differ from that of materials collected from diverse geographical regions. We noticed that wheat accessions collected from Asia formed a cluster distinct from those of other European and Australian accessions, suggesting that accessions might have adapted to the local environment or have been bred for specific objectives. Similarly, Muqaddasi’s study [69] reported that spring wheat accessions originating from Asia were distinguished from European accessions by PCA, and a similar pattern was also detected in [70] in which wheat varieties from the same origins clustered together.

### LD in diverse wheat species

LD between two independent loci is known to be affected by mutation, effective population size, mating system, gene flow, genetic drift, and selection [71]; therefore, the rate of LD decay can vary between subgenomes, species or analyzed populations. In our study, among the three species, bread wheat had the most rapid genome-wide LD decay; specifically, the A genome decayed the most rapidly, followed by the D genome and B genome. The fastest LD decay in the A genome has been reported in several studies [33, 72].

Spelt wheat was estimated to diverge from modern bread wheat several thousand years ago; interestingly, the genome-wide LD decay patterns in the A and B genomes (but not the D genome) were quite similar between the two hexaploid wheat lines, where *r*^*2*^ = 0.2 occurred at 21.08 Mb as opposed to 1.67 Mb in bread wheat. Such long-range LD decay was also observed in 293 Swiss spelt wheat and 123 European spelt accessions [73, 74]. Würschum’s results [73] suggested that the results obtained should be treated with caution due to the lower marker density in the D genome compared with the other genomes. In our study, the number of polymorphic markers in the D genome was four times lower in the 60 spelt wheat accessions compared with the 765 bread wheat accessions; however, considering that the number of polymorphic markers in the A or B genome in the 60 spelt wheat accessions was three times lower than that in the 765 bread wheat accessions, the LD pattern was not dramatically different; as such, we suspect that other factors contributed to the extended LD of the D genome in spelt wheat. It is widely accepted that the D genome is derived from diploid *Aegilops tauschii* and is the youngest genome in hexaploid wheat [7, 10, 75, 76]. It is possible that the D genome in spelt wheat has not accumulated a significant number of mutations or recombination events that could contribute to the degree of LD decay.

### Association between grain traits and SNP markers

Wheat chromosome 7A is known to harbor genes related to grain traits, among these genes, *WAPO-A1* was shown to colocalize with the QTL region identified in this study and was identified as a candidate gene for a QTL responsible for spikelet number per spike [60]. Another significant SNP detected in the present work, Kukri_c2912_2029, was found on chromosome 2A (S6.1-Grain length in Supplementary Table S6) in the vicinity of *TaGW7*, which regulates grain length and grain width [77], and the significant SNP marker BS00073670_51, on chromosome 5A (S7.1-Grain length in Supplementary Table S7), was found near *TaGL3-5A*, which is associated with grain length and thousand-kernel weight [78]. Co-localization of significant SNPs on chromosome 7A associated with grain surface area, grain perimeter, and grain length was found in this study (Supplementary Fig. S9, S10 and S11). The phenotypic correlation between these traits is high (Pearson correlation coefficient ranges between 0.53–0.95), however traits distribution varies (Supplementary Fig. S12), this suggested the candidate gene in the QTL region on chromosome 7A could have different effects, highlighting the importance to identify the causal genes or variants controlling grain traits. Rice genes controlling grain morphological traits have been studied extensively [53], wheat genes *TaGW7* and *TaGL3-5A* were found to be orthologous to the rice *GW7* [79, 80] and *GL3.1* [63, 81]. Another important trait determining wheat quality is grain color. The long arm of chromosome 3B is known to carry a grain color locus “*R*” [82], a candidate gene “*Tamyb10-B1*” encoded R2R3-type MYB domain protein was further investigated for its allelic diversity and effect on grain color [83], *Tamyb10-B1* gene was found to locate in the LD block calculated from the significant associated-SNPs detected in this study (Supplementary Fig. 13). The agreement between our GWAS results and those concerning previously identified genes governing grain-related traits not only validated the SNP quality resulting from our in-house pipeline but also suggested that diagnostic markers for grain traits could be potentially developed by the use of 90 K SNP markers. Wheat breeders could identify the elite germplasm carrying a beneficial allele for the trait of interest from this study. Pyramiding several QTLs in the target variety’s background through diligent crossing or inter-mating and marker-assisted selection could improve yield or quality significantly.

## Conclusions

With the recent release of IWGSC RefSeq v2.1 in 2021, this study reported an updated and accurate physical position of 57,398 SNP loci on a high-density 90 K wheat genotyping array. Using the information gained in this work, we anticipate that wheat researchers who previously applied 90 K array markers for QTL mapping can seamlessly update their findings to RefSeq v2.1. In addition, the diverse wheat germplasms analyzed in this work constitute great resources for investigating population differentiation between and within hexaploid wheat accessions, and the genetic variation inherent within each population reflects its evolutionary and breeding history. Finally, considering our materials and genotypes are available to the public, we anticipate more QTLs or trait-linked SNP markers will be discovered in different environmental conditions, which could also increase our understanding of genetic architecture controlling quantitative traits.

## Supplementary Information

**Additional file 1: Supplementary Fig. S1.** The distribution of 583 markers revealed inconsistent chromosomal assignments between the two genome assemblies. The left hemisphere colored light blue represents IWGSC RefSeq v2.1, and the right hemisphere colored pink indicates IWGSC RefSeq v1.0. The orientation in IWGSC RefSeq v2.1 is counterclockwise, and that in IWGSC RefSeq v1.0 is clockwise. Each line in the middle of the Circos plot connects the physical positions in the IWGSC RefSeq v1.0 and v2.1 reference genomes of each marker. The colors of these lines are assigned according to which homoeologous group each marker belongs to in IWGSC RefSeq v1.0.

**Additional file 2: Supplementary Fig. S2.** Distribution of markers with the same chromosomal assignment but different orientations or order between IWGSC RefSeq v1.0 and IWGSC RefSeq v2.1. The x axis indicates wheat chromosomes, and the y axis shows the physical position along the chromosome. The chromosomes of IWGSC RefSeq v1.0 are shown in black, and chromosomes of IWGSC RefSeq v2.1 are colored blue. The red line connects the physical positions of each marker between the two RefSeq assemblies.

**Additional file 3: Supplementary Fig. S3.** PCA of 765 bread wheat accessions on the basis of 29,803 SNPs in the A, B, and D genomes. The proportion of the total variance explained by each PC is shown on the axis label. The color of each point indicates the subpopulation assigned based on an ADMXITURE analysis.

**Additional file 4: Supplementary Fig. S4.** Genome-wide scan for selection signal between two subpopulations, Pop1 and Pop2. The points colored blue indicate outliers detected according to a significance threshold of a FDR < 0.05.

**Additional file 5: Supplementary Fig. S5.** LD in subpopulations Pop1 and Pop2. (a) LD decay at the whole-genome level. (b) LD decay of subgenome A. (c) LD decay of subgenome B. (d) LD decay of subgenome D. The physical distance (in megabases) is plotted against the LD estimate (*r*^2^) for pairs of markers.

**Additional file 6: Supplementary Fig. S6.** Genome-wide association mapping for grain width. Manhattan plots of the four models (GLM, GLM_PC, MLM, MLM_PC) and associated quantile-quantile (Q-Q) plots representing the statistical association between each SNP and grain width.

**Additional file 7: Supplementary Fig. S7.** Genome-wide association mapping for the grain perimeter. Manhattan plots of the four models (GLM, GLM_PC, MLM, MLM_PC) and associated quantile-quantile (Q-Q) plots representing the statistical association between each SNP and grain perimeter.

**Additional file 8: Supplementary Fig. S8.** Genome-wide association mapping for grain area. Manhattan plots of the four models (GLM, GLM_PC, MLM, MLM_PC) and associated quantile-quantile (Q-Q) plots representing the statistical association between each SNP and grain area.

**Additional file 9: Supplementary Fig. S9.** Genome-wide association mapping for color channel 1. Manhattan plots of the four models (GLM, GLM_PC, MLM, MLM_PC) and associated quantile-quantile (Q-Q) plots representing the statistical association between each SNP and color channel 1.

**Additional file 10: Supplementary Fig. S10.** Genome-wide association mapping for color channel 2. Manhattan plots of the four models (GLM, GLM_PC, MLM, MLM_PC) and associated quantile-quantile (Q-Q) plots representing the statistical association between each SNP and color channel 2.

**Additional file 11: Supplementary Fig. S11.** Genome-wide association mapping for color channel 3. Manhattan plots of the four models (GLM, GLM_PC, MLM, MLM_PC) and associated quantile-quantile (Q-Q) plots representing the statistical association between each SNP and color channel 3.

**Additional file 12: Supplementary Fig. S12.** Phenotypic distribution of six grain traits and correlation analysis.

**Additional file 13: Supplementary Fig. S13.** LD heatmap of the grain color locus. The pairwise LD of significant SNPs associated with three color channels on chromosome 3B was calculated and plotted. The position of significant SNPs and *Tamyb10-B1* gene was labeled.

**Additional file 14.**

**Additional file 15.**

**Additional file 16.**

**Additional file 17.**

**Additional file 18.**

**Additional file 19.**

**Additional file 20.**

**Additional file 21.**

## Data Availability

The genotype dataset generated and analyzed in this research are included within the article and its supplementary files. All wheat accessions used in this study are housed in Taichung District Agricultural Research and Extension Station (TDAIS) seed bank and available on reasonable request. These accessions were originally collected from USDA and CIMMYT genebanks, USDA PI number and CIMMYT number are listed in the Supplementary Table S1. The authors declare that all that permissions or licenses were obtained to collect the wheat germplasm from public depositories.
