# Supplementary figures and images for: Patterns of genetic variation and QTLs controlling grain traits in a collection of global wheat germplasm revealed by high-quality SNP markers
Source: BMC Plant Biol. 2022 Sep 22;22:455. doi: 10.1186/s12870-022-03844-x (PMC9494784; doi:10.1186/s12870-022-03844-x)

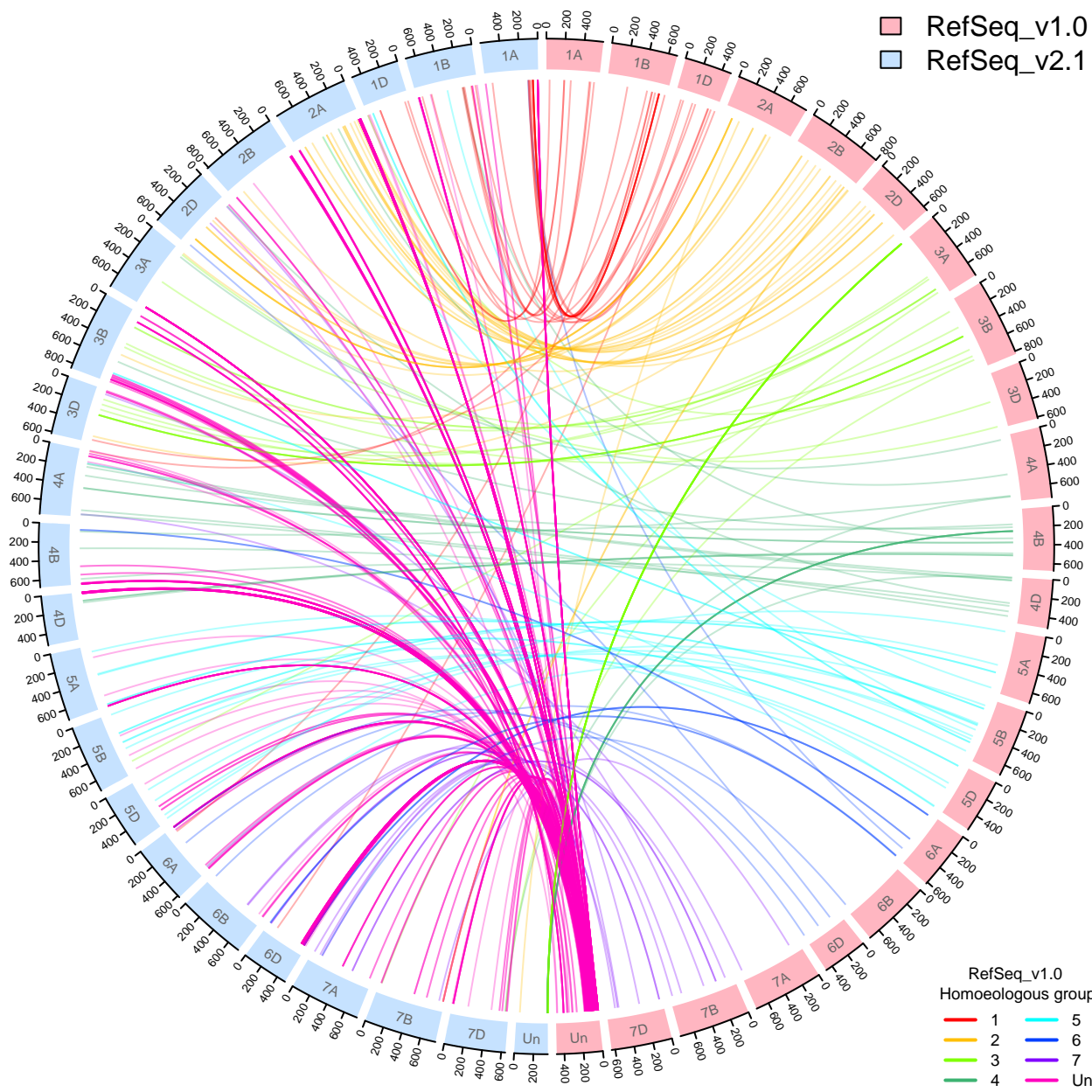

Supplement: Supplementary file 1 — Additional file 1: Supplementary Fig. S1. The distribution of 583 markers revealed inconsistent chromosomal assignments between the two genome assemblies. The left hemisphere colored light blue represents IWGSC RefSeq v2.1, and the right hemisphere colored pink indicates IWGSC RefSeq v1.0. The orientation in IWGSC RefSeq v2.1 is counterclockwise, and that in IWGSC RefSeq v1.0 is clockwise. Each line in the middle of the Circos plot connects the physical positions in the IWGSC RefSeq v1.0 and v2.1 reference genomes of each marker. The colors of these lines are assigned according to which homoeologous group each marker belongs to in IWGSC RefSeq v1.0. [file 12870_2022_3844_MOESM1_ESM.pdf]

Physical Map

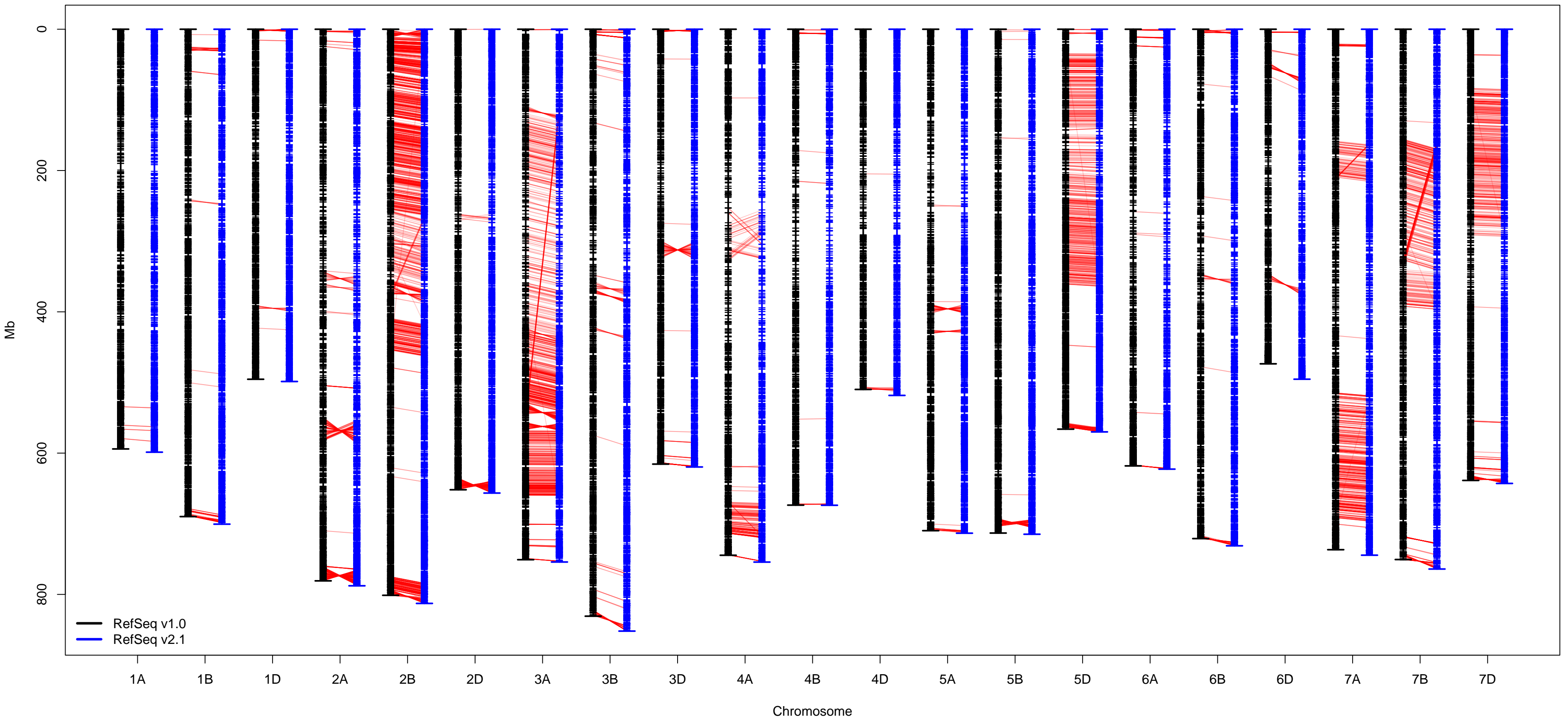

Supplement: Supplementary file 2 — Additional file 2: Supplementary Fig. S2. Distribution of markers with the same chromosomal assignment but different orientations or order between IWGSC RefSeq v1.0 and IWGSC RefSeq v2.1. The x axis indicates wheat chromosomes, and the y axis shows the physical position along the chromosome. The chromosomes of IWGSC RefSeq v1.0 are shown in black, and chromosomes of IWGSC RefSeq v2.1 are colored blue. The red line connects the physical positions of each marker between the two RefSeq assemblies. [file 12870_2022_3844_MOESM2_ESM.pdf]

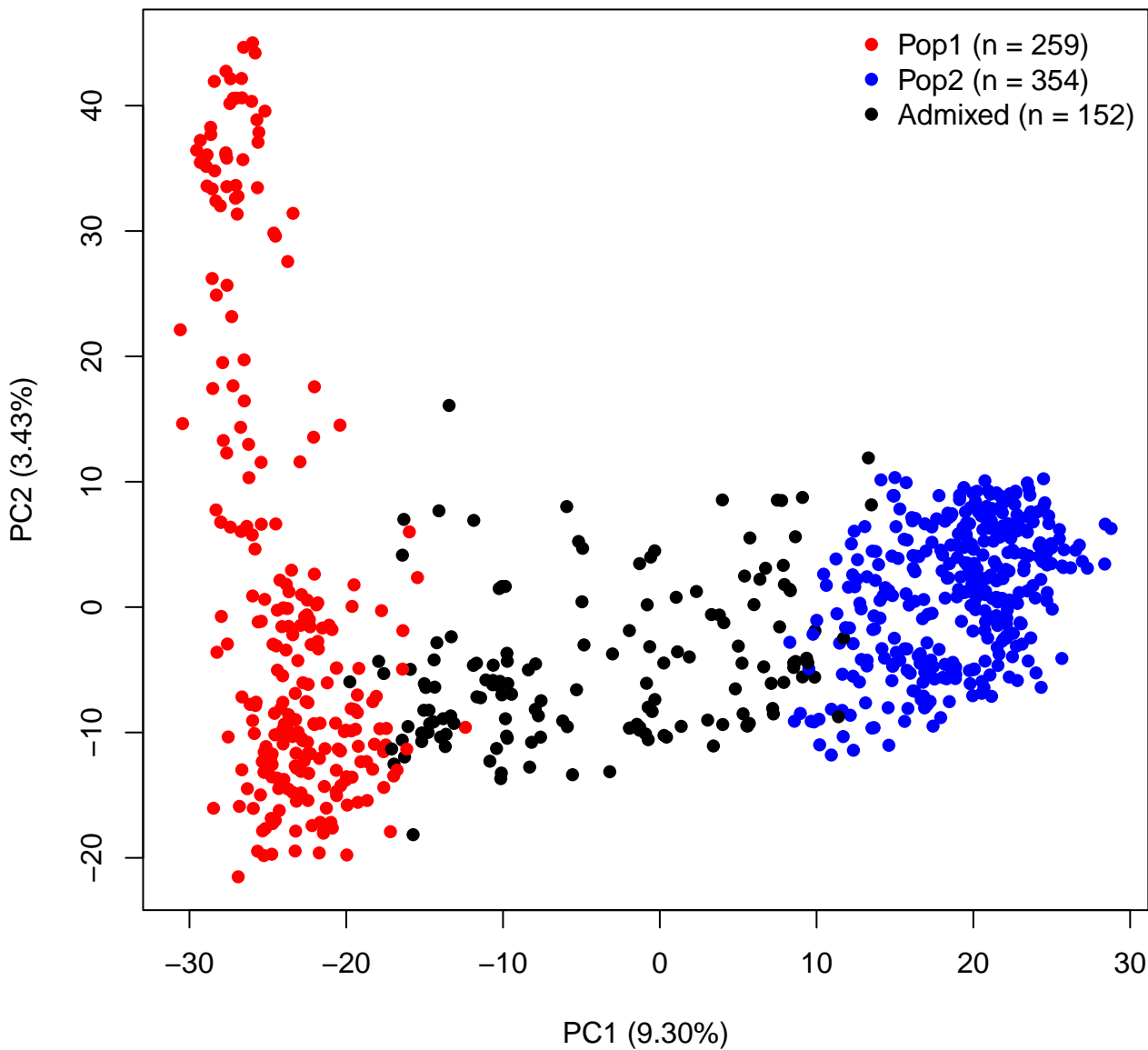

Supplement: Supplementary file 3 — Additional file 3: Supplementary Fig. S3. PCA of 765 bread wheat accessions on the basis of 29,803 SNPs in the A, B, and D genomes. The proportion of the total variance explained by each PC is shown on the axis label. The color of each point indicates the subpopulation assigned based on an ADMXITURE analysis. [file 12870_2022_3844_MOESM3_ESM.pdf]

Pop1 vs Pop2

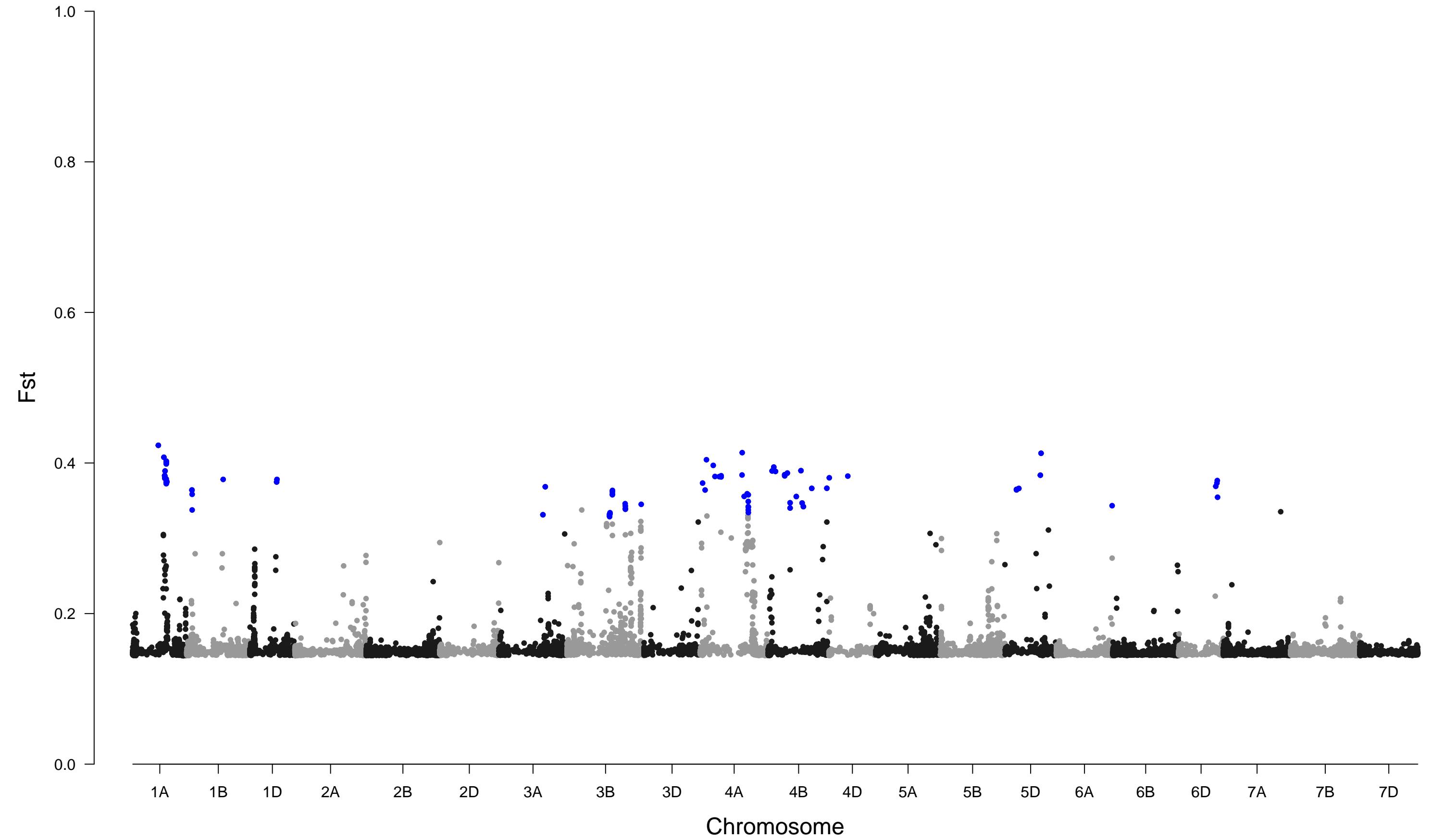

Supplement: Supplementary file 4 — Additional file 4: Supplementary Fig. S4. Genome-wide scan for selection signal between two subpopulations, Pop1 and Pop2. The points colored blue indicate outliers detected according to a significance threshold of a FDR < 0.05. [file 12870_2022_3844_MOESM4_ESM.pdf]

**a**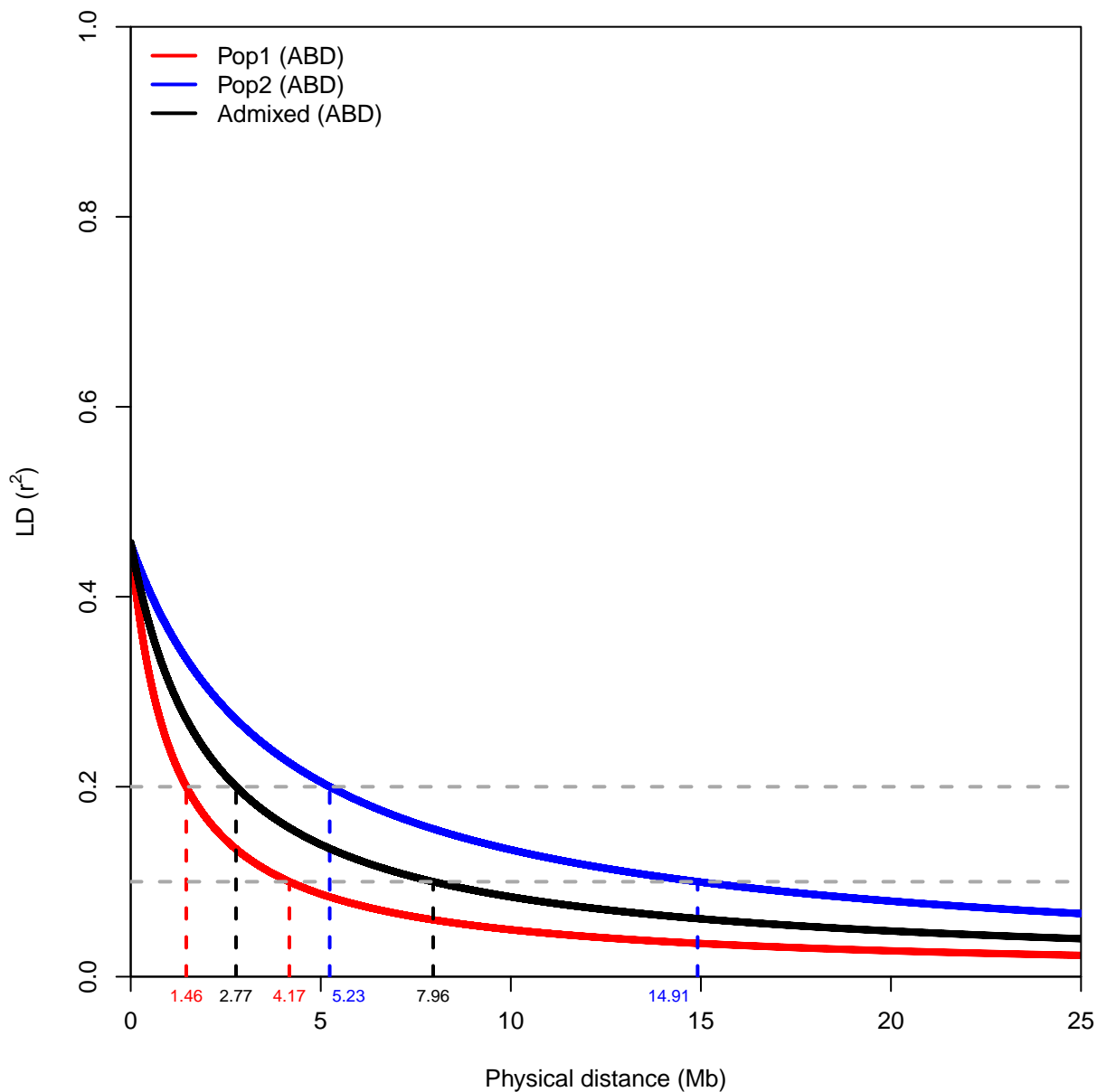**b**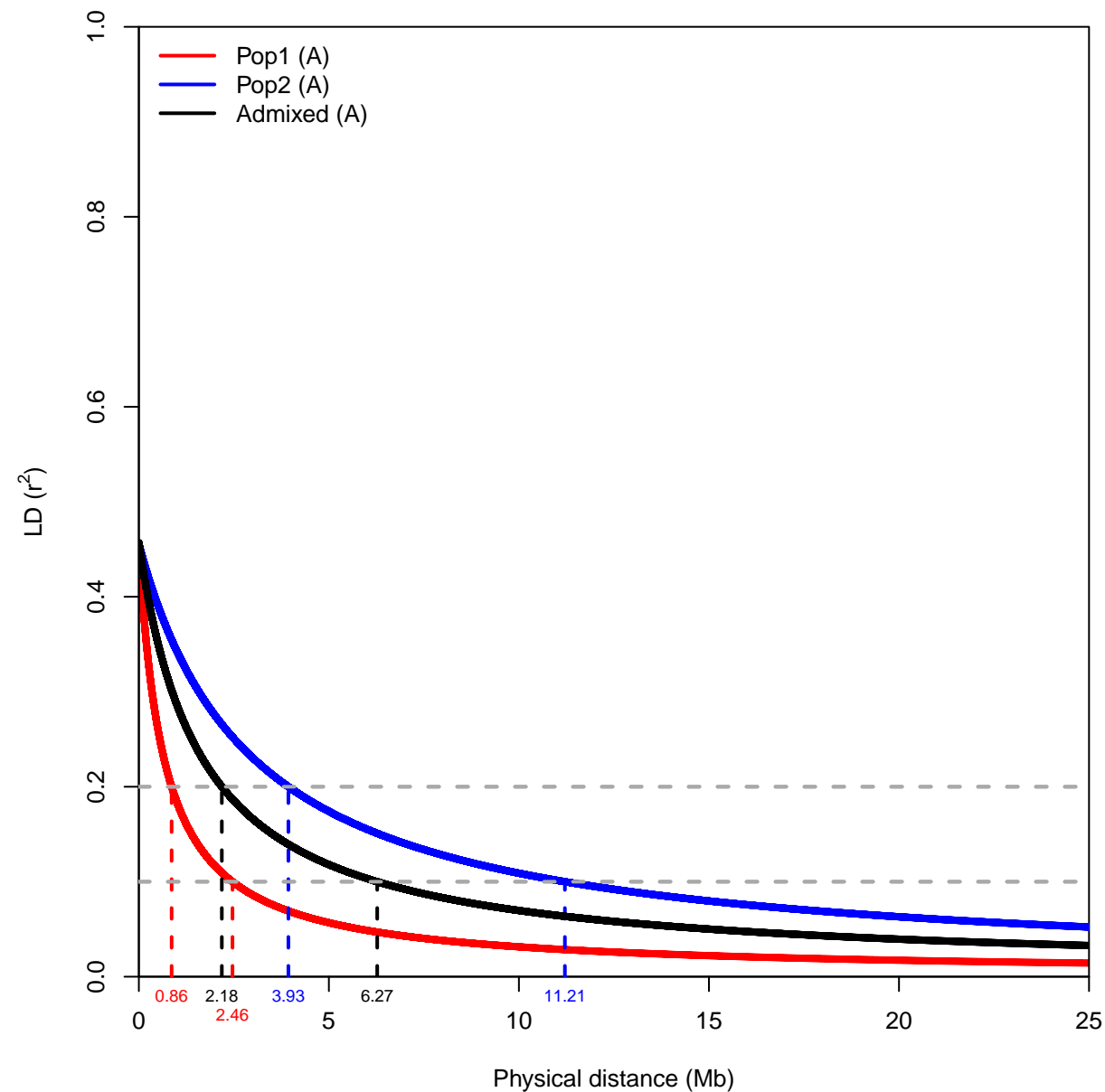**c**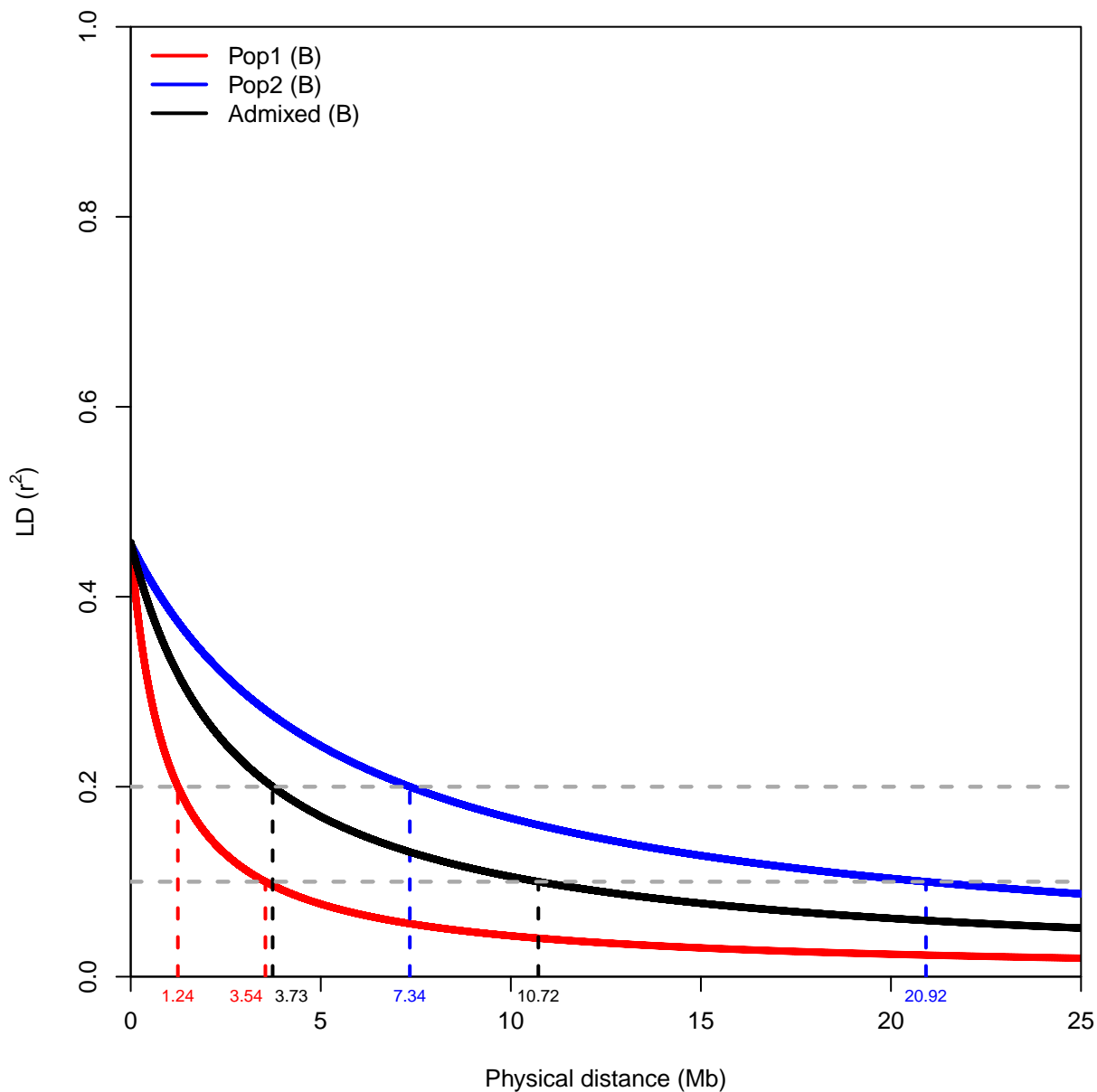**d**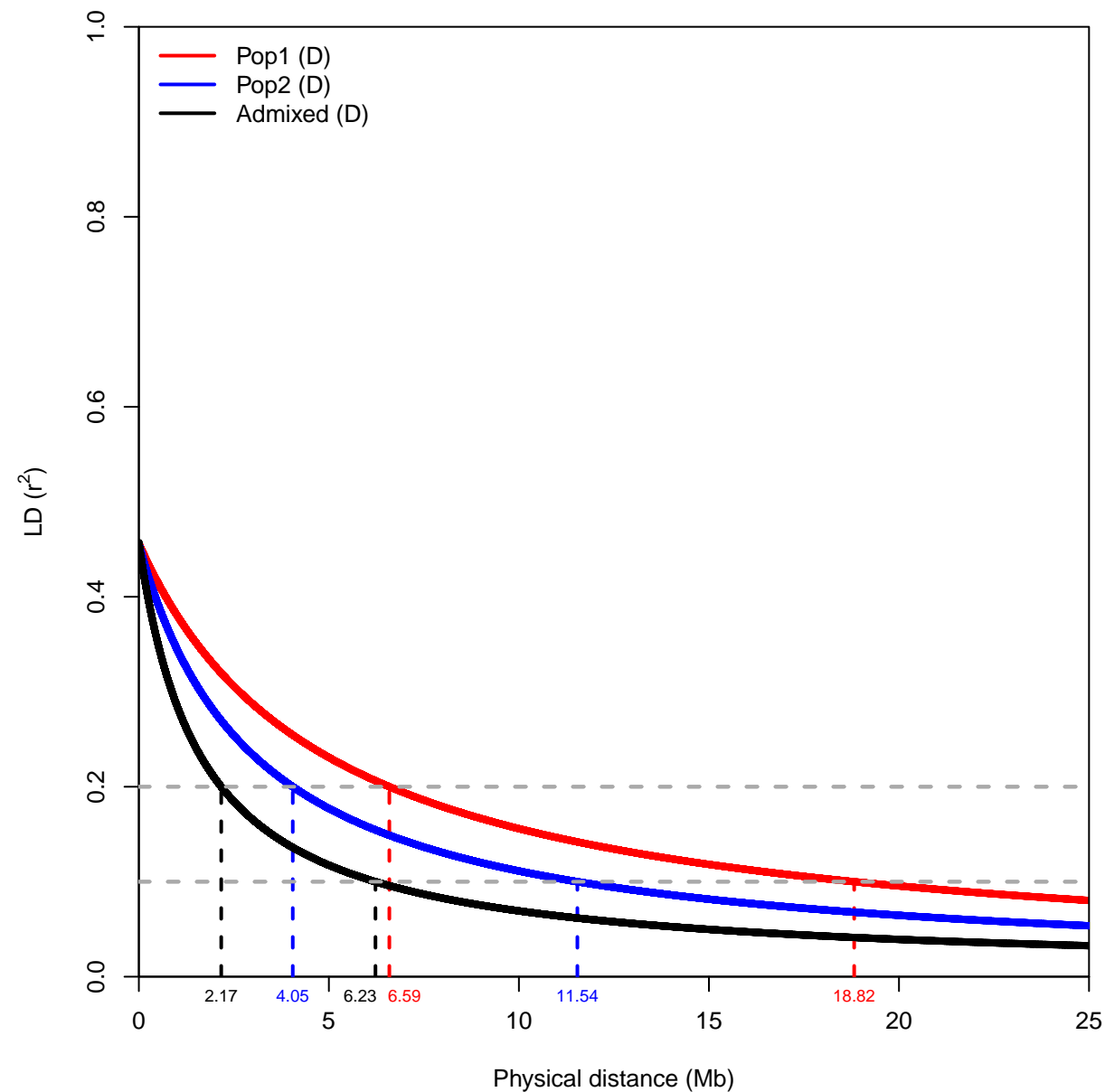

Supplement: Supplementary file 5 — Additional file 5: Supplementary Fig. S5. LD in subpopulations Pop1 and Pop2. (a) LD decay at the whole-genome level. (b) LD decay of subgenome A. (c) LD decay of subgenome B. (d) LD decay of subgenome D. The physical distance (in megabases) is plotted against the LD estimate (r2) for pairs of markers. [file 12870_2022_3844_MOESM5_ESM.pdf]

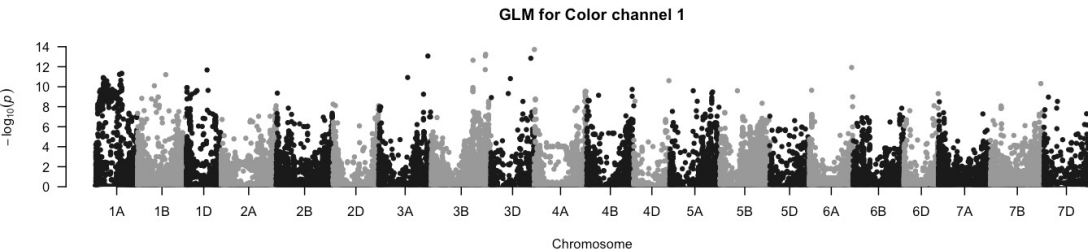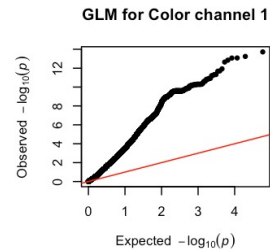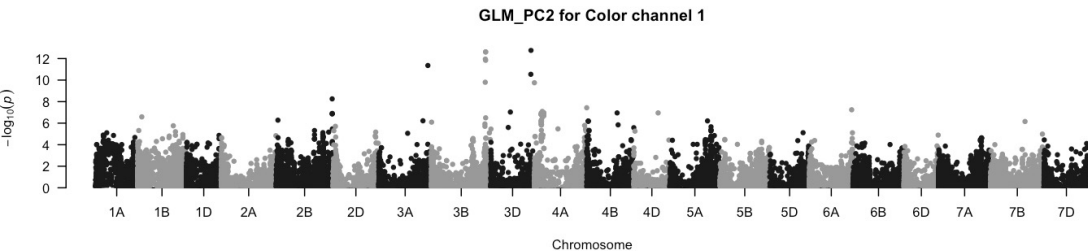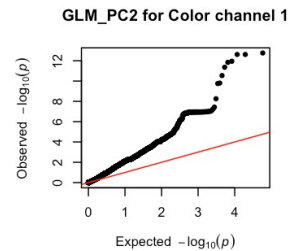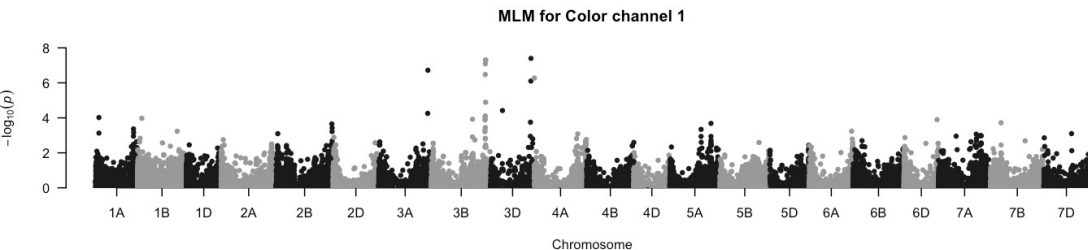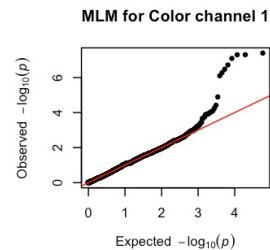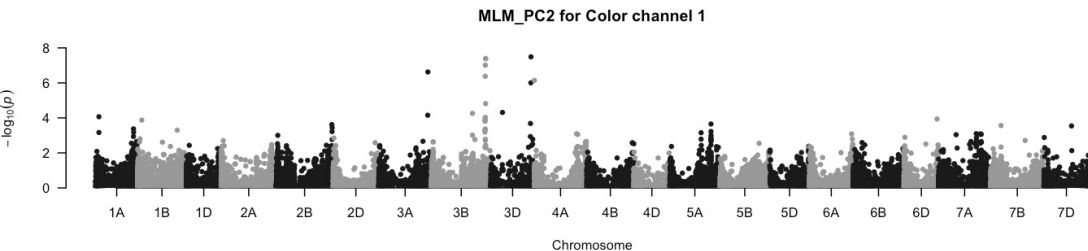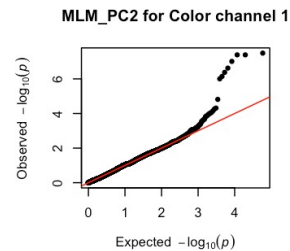

Supplement: Supplementary file 9 — Additional file 9: Supplementary Fig. S9. Genome-wide association mapping for color channel 1. Manhattan plots of the four models (GLM, GLM_PC, MLM, MLM_PC) and associated quantile-quantile (Q-Q) plots representing the statistical association between each SNP and color channel 1. [file 12870_2022_3844_MOESM9_ESM.pdf]

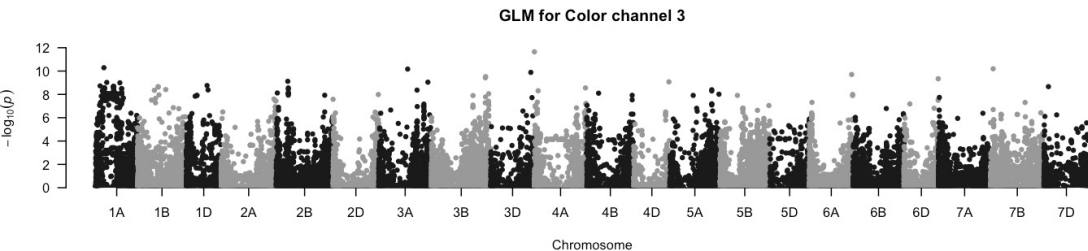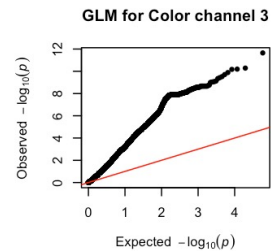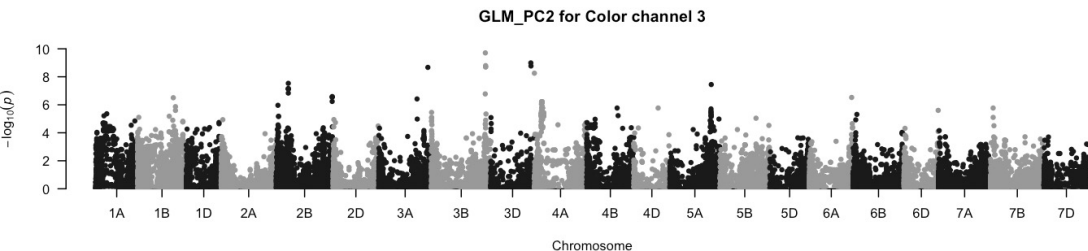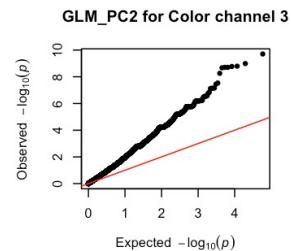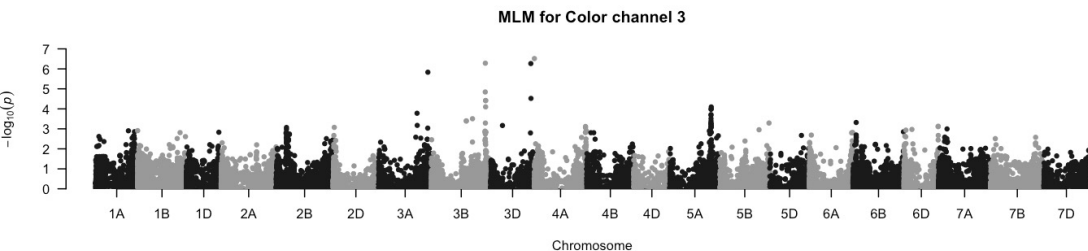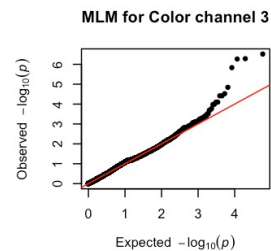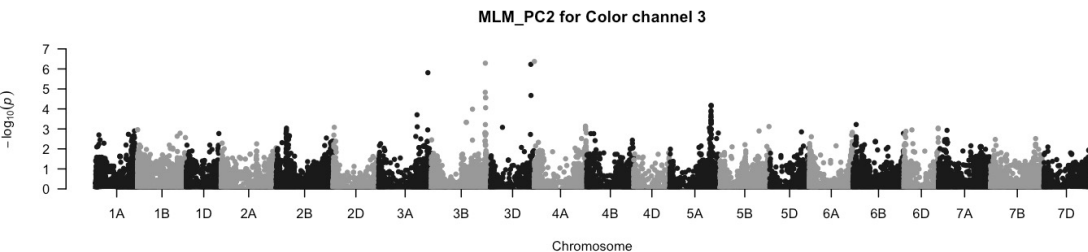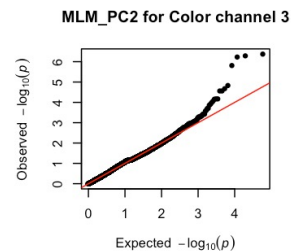

Supplement: Supplementary file 11 — Additional file 11: Supplementary Fig. S11. Genome-wide association mapping for color channel 3. Manhattan plots of the four models (GLM, GLM_PC, MLM, MLM_PC) and associated quantile-quantile (Q-Q) plots representing the statistical association between each SNP and color channel 3. [file 12870_2022_3844_MOESM11_ESM.pdf]

(A)

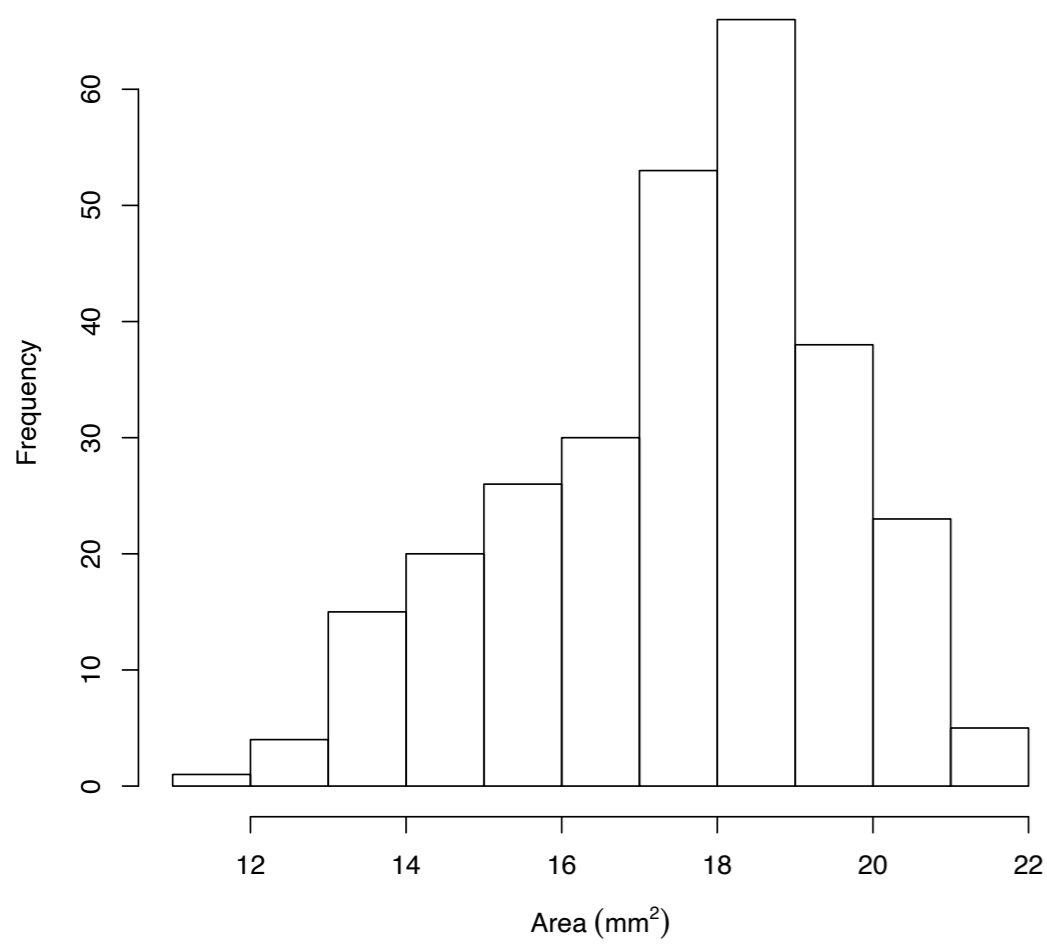

(B)

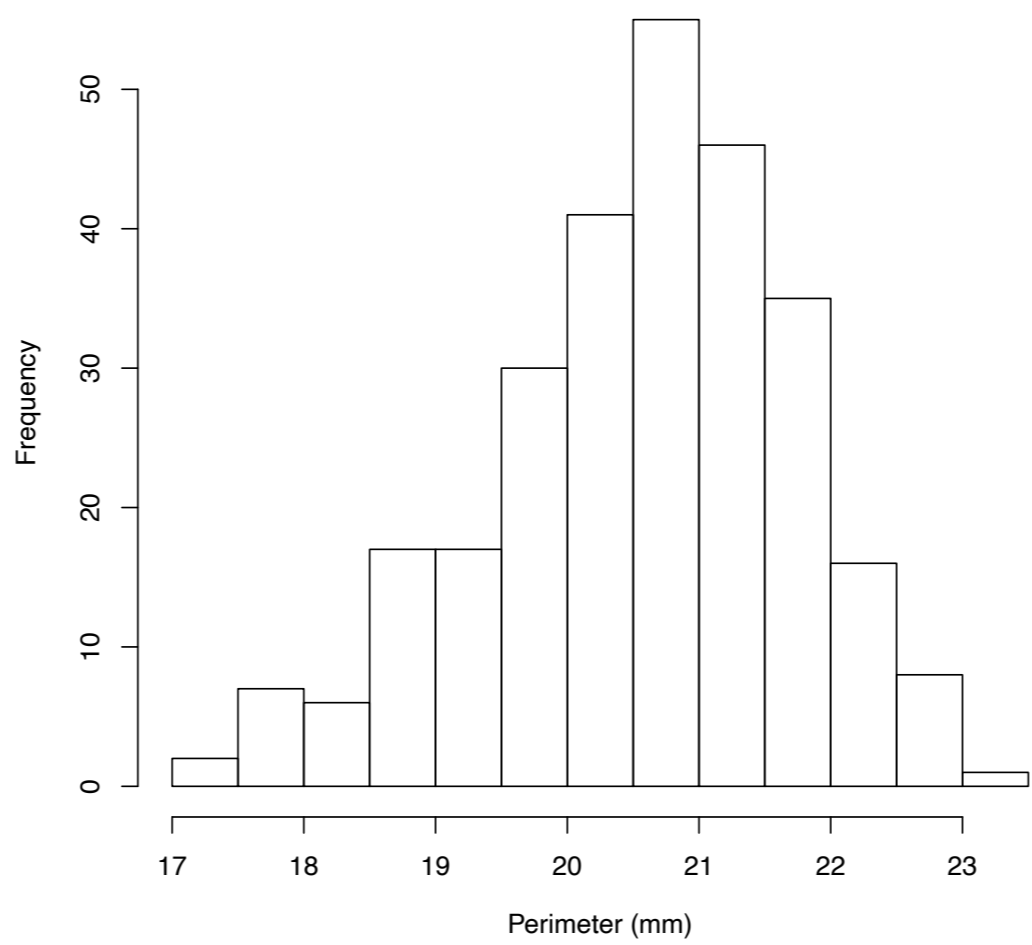

(C)

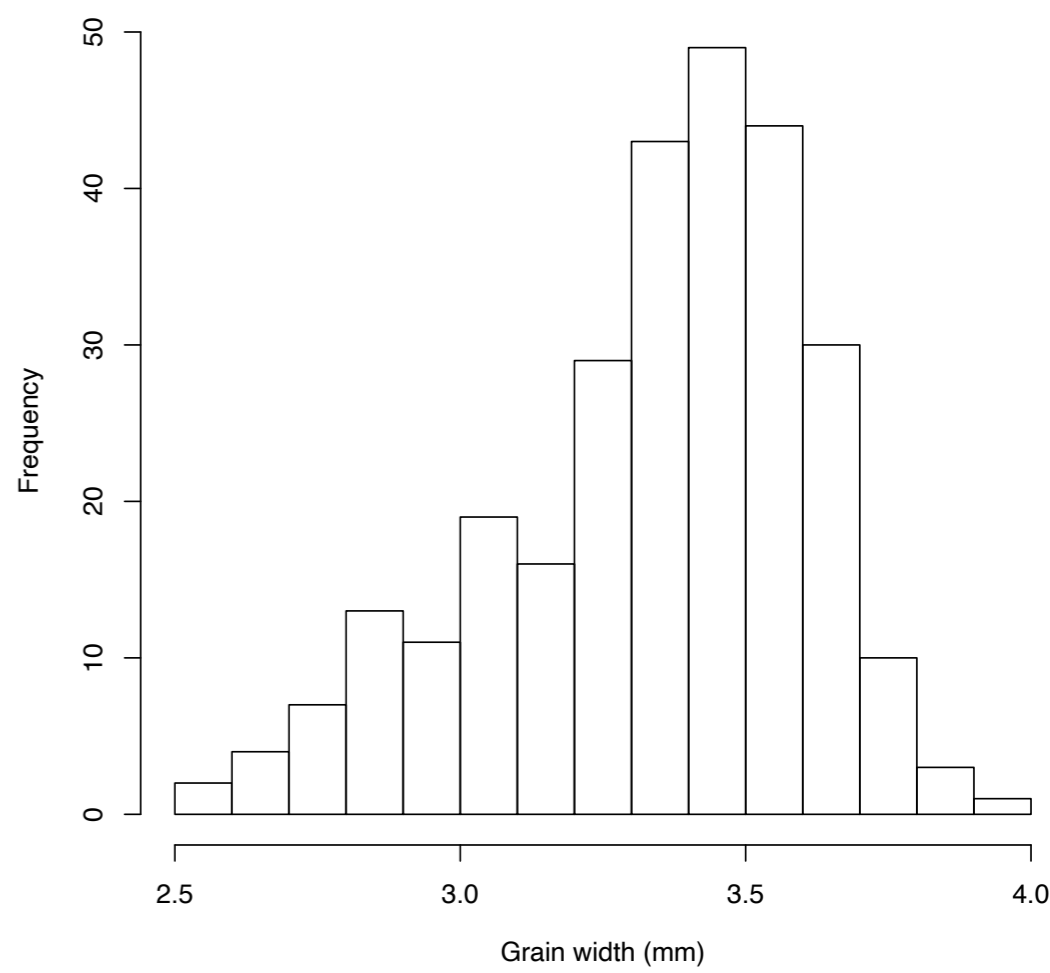

(D)

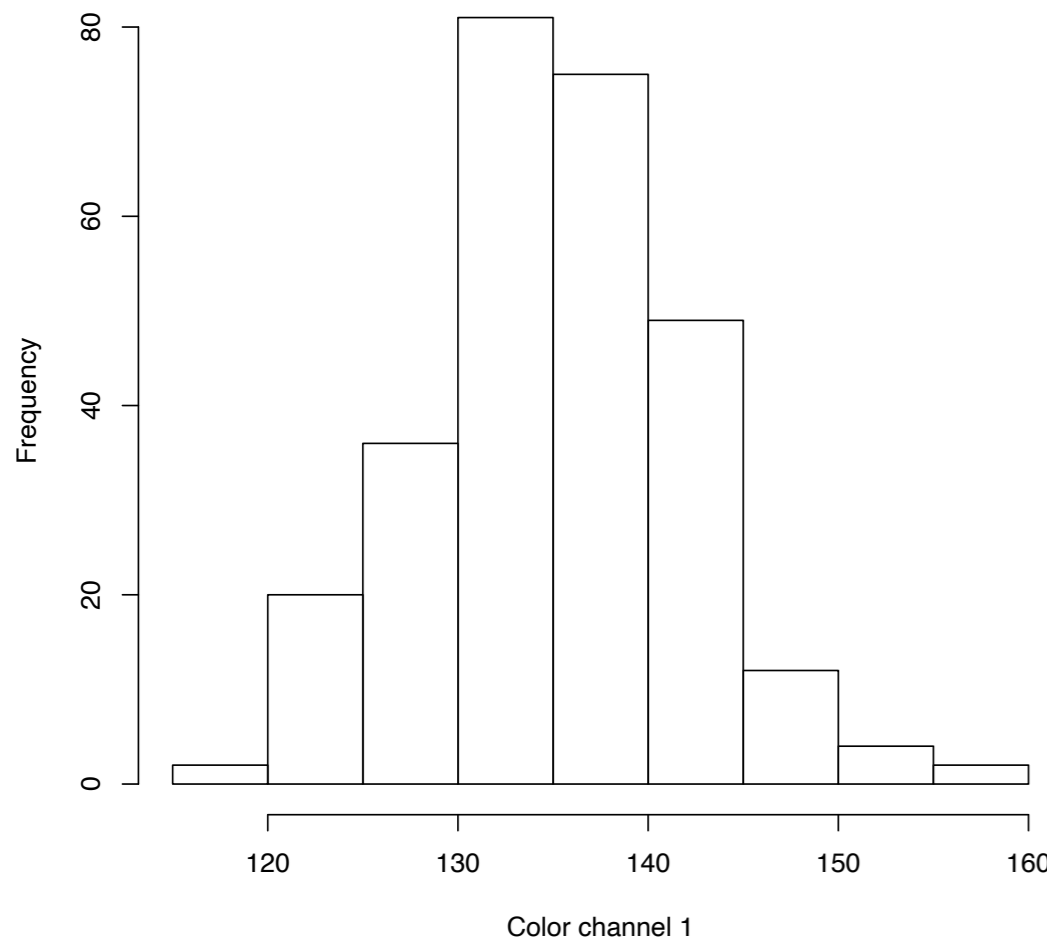

(E)

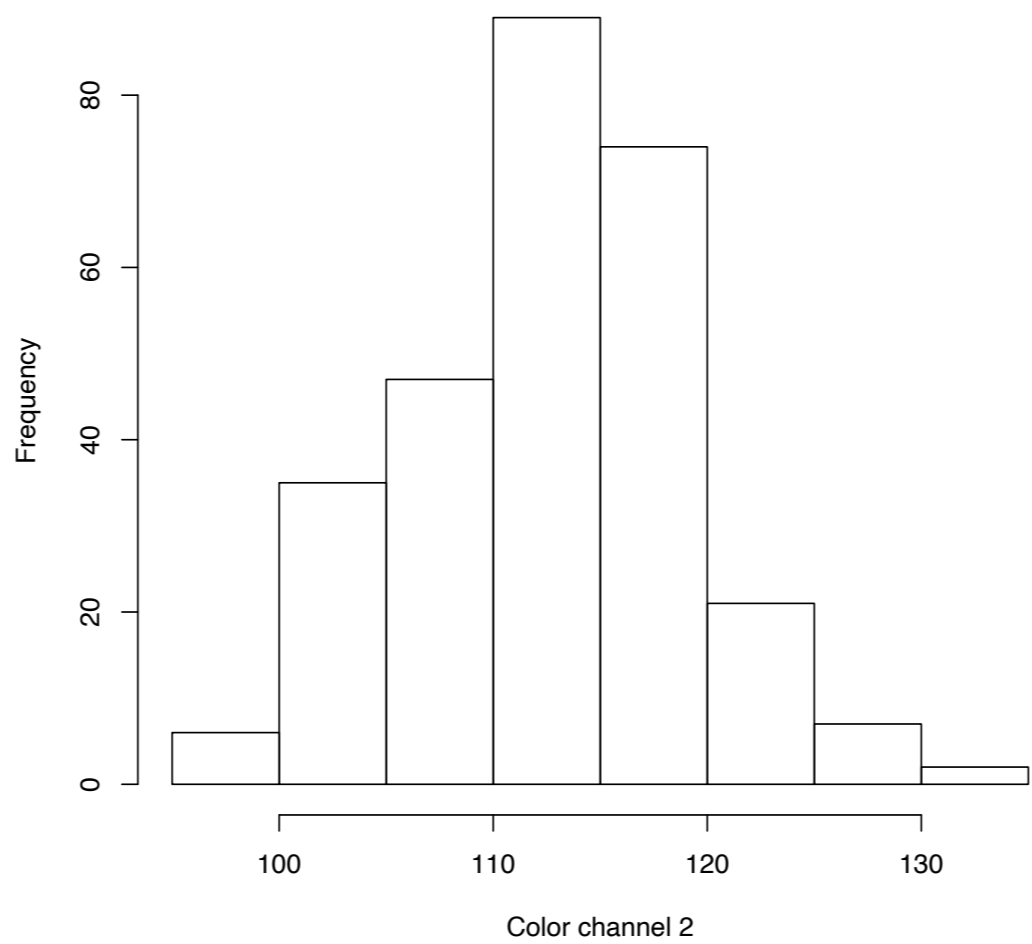

(F)

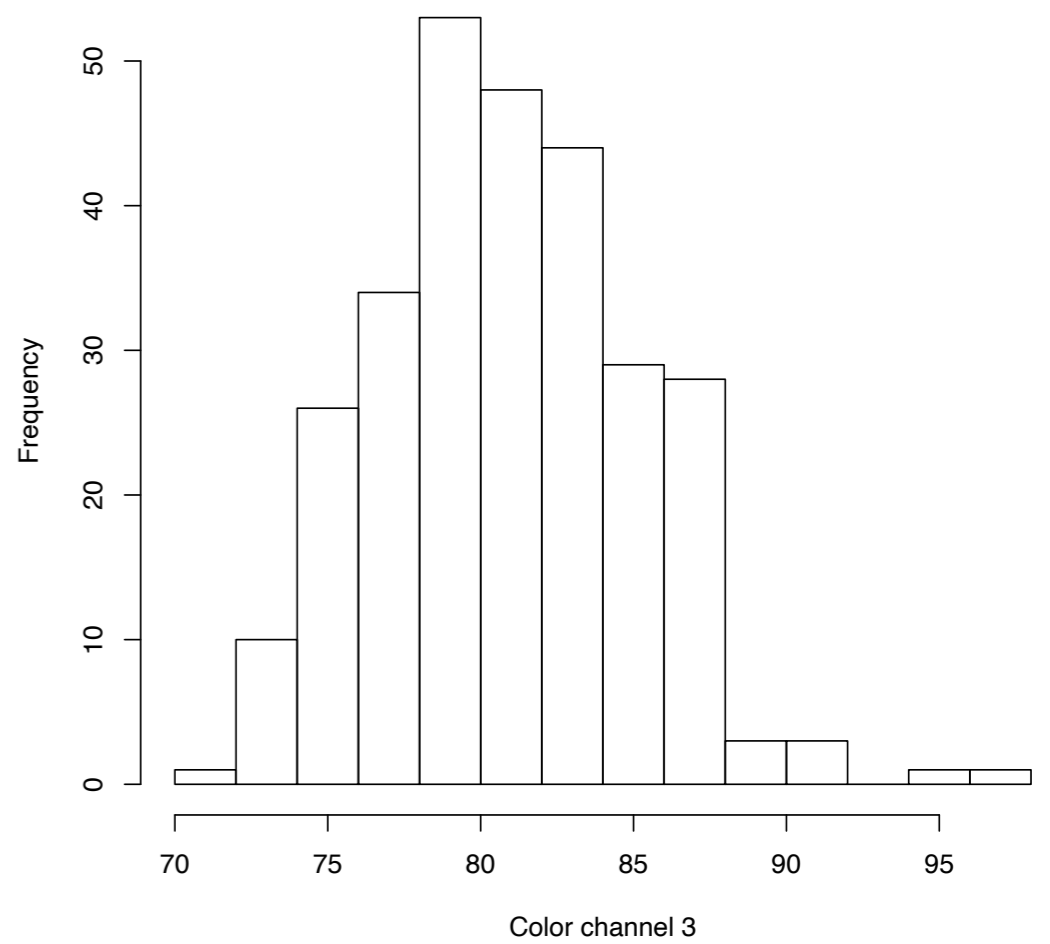

(G)

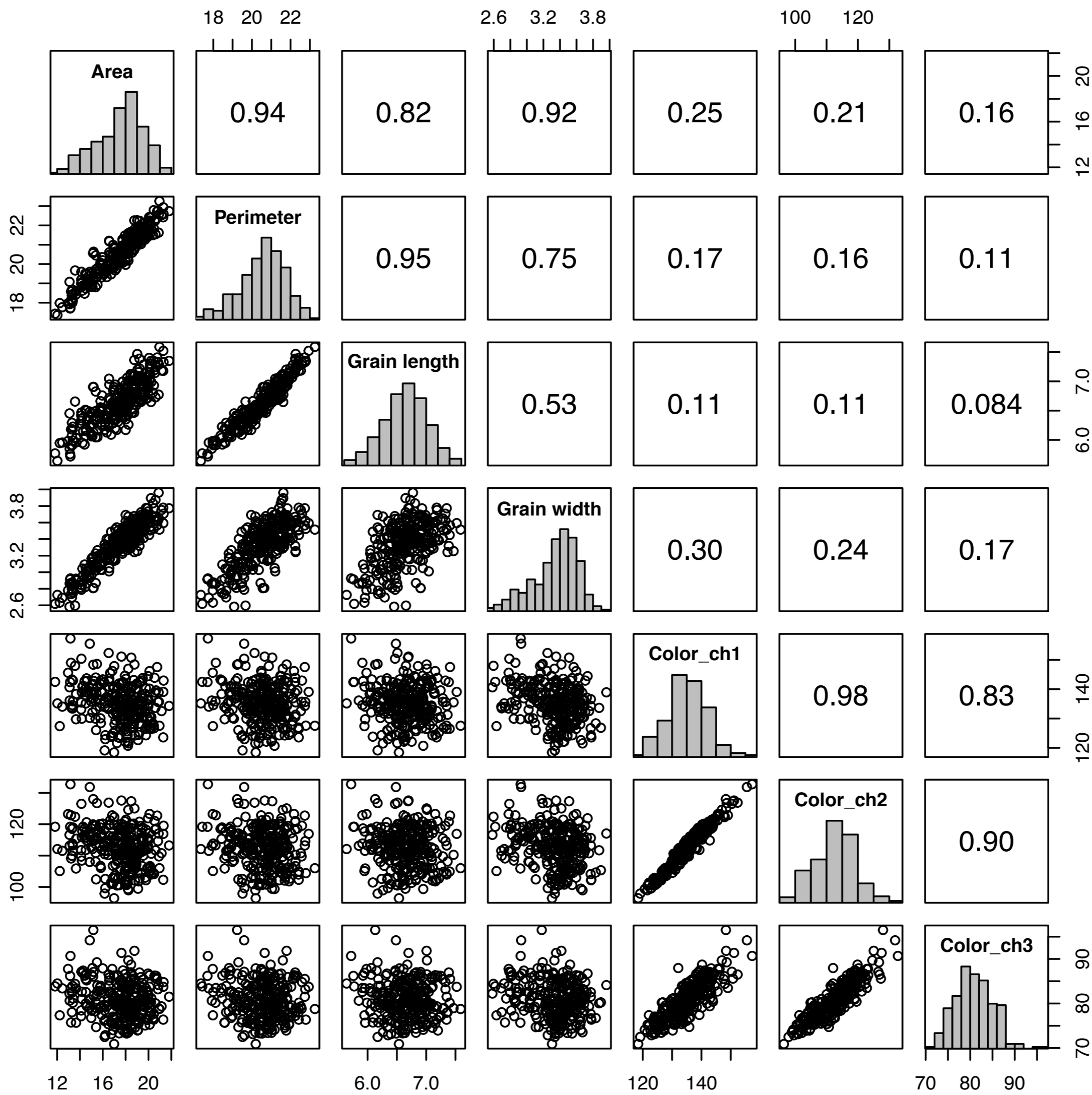

Supplement: Supplementary file 12 — Additional file 12: Supplementary Fig. S12. Phenotypic distribution of six grain traits and correlation analysis. [file 12870_2022_3844_MOESM12_ESM.pdf]

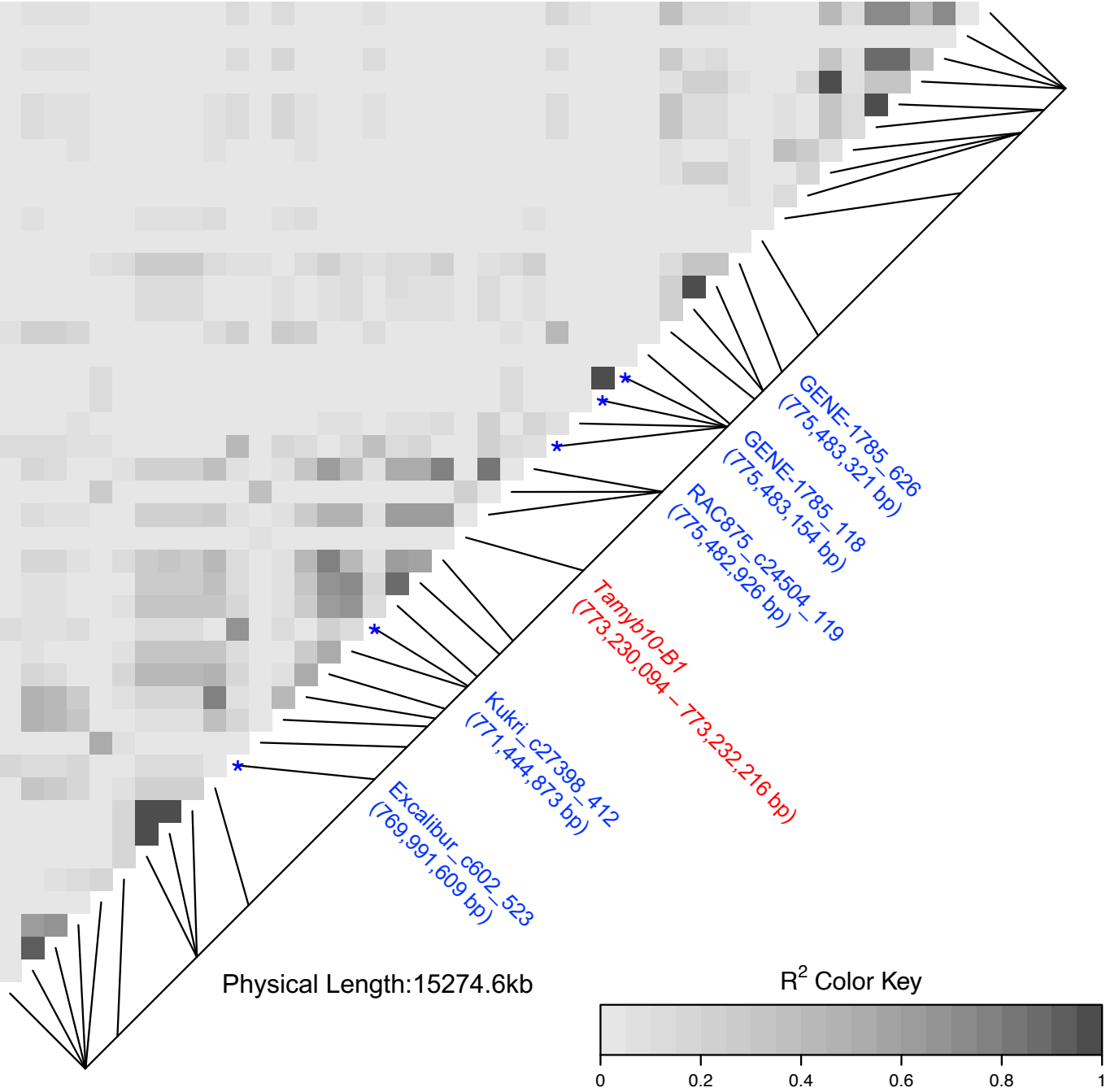

Supplement: Supplementary file 13 — Additional file 13: Supplementary Fig. S13. LD heatmap of the grain color locus. The pairwise LD of significant SNPs associated with three color channels on chromosome 3B was calculated and plotted. The position of significant SNPs and Tamyb10-B1 gene was labeled. [file 12870_2022_3844_MOESM13_ESM.pdf]
