# Supplementary material for: Patterns of genetic variation and QTLs controlling grain traits in a collection of global wheat germplasm revealed by high-quality SNP markers
Source: BMC Plant Biol. 2022 Sep 22;22:455. doi: 10.1186/s12870-022-03844-x (PMC9494784; doi:10.1186/s12870-022-03844-x)
Supplement: Supplementary file 10 — Additional file 10: Supplementary Fig. S10. Genome-wide association mapping for color channel 2. Manhattan plots of the four models (GLM, GLM_PC, MLM, MLM_PC) and associated quantile-quantile (Q-Q) plots representing the statistical association between each SNP and color channel 2. [file 12870_2022_3844_MOESM10_ESM.pdf]

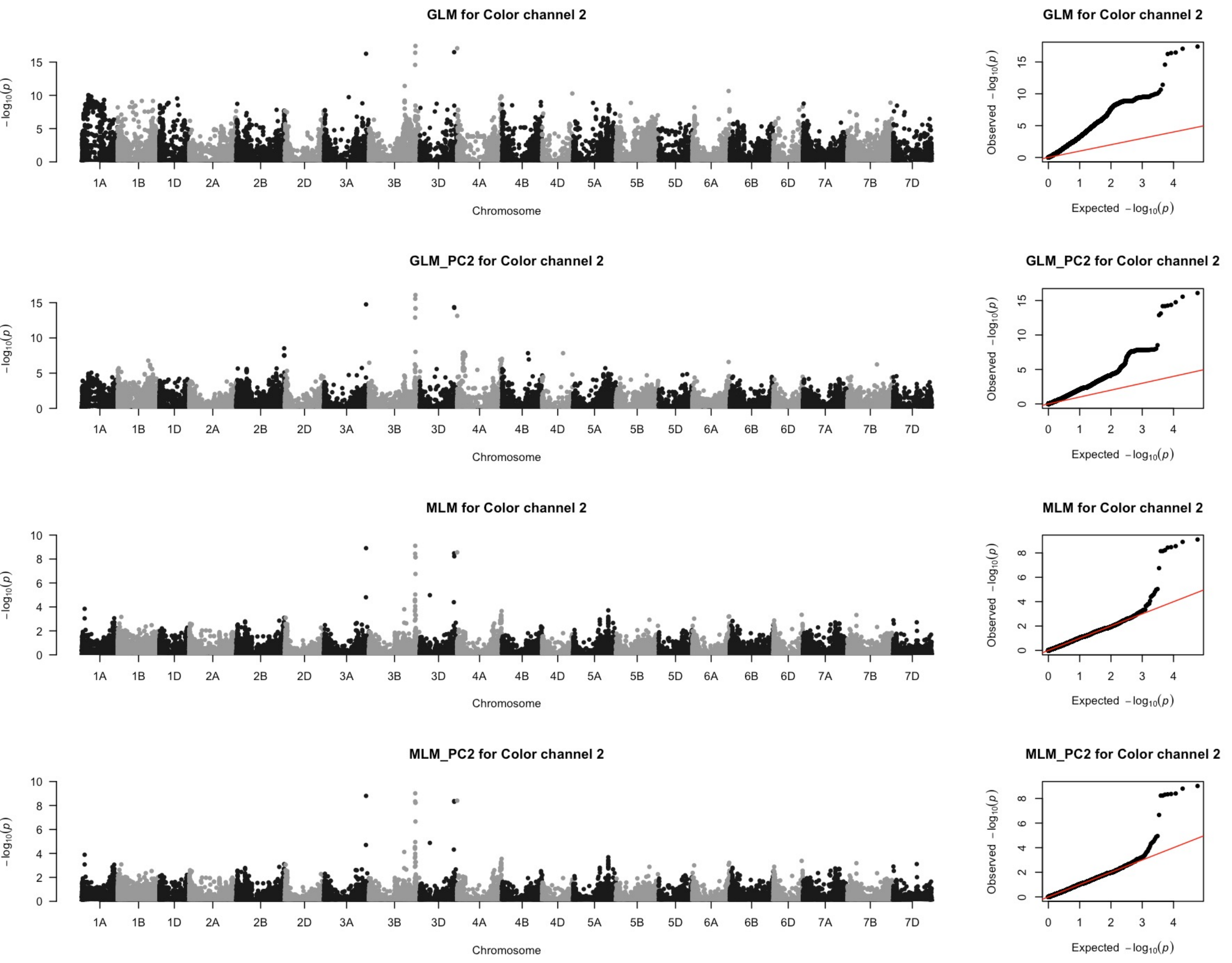
GLM for Color channel 2
GLM_PC2 for Color channel 2
MLM for Color channel 2
MLM_PC2 for Color channel 2
$-\log_{10}(p)$
Chromosome
1A 1B 1D 2A 2B 2D 3A 3B 3D 4A 4B 4D 5A 5B 5D 6A 6B 6D 7A 7B 7D
Observed $-\log_{10}(p)$
Expected $-\log_{10}(p)$
